# Supplementary material for: Comprehensive benchmarking of tools for nanopore-based detection of DNA methylation
Source: Nat Commun. 2026 Jul 15;17:8685. doi: 10.1038/s41467-026-75183-6 (PMC13490465; doi:10.1038/s41467-026-75183-6)
Supplement: Supplementary file 1 [file 41467_2026_75183_MOESM1_ESM.pdf]

Supplementary Figures for

Comprehensive benchmarking of tools for nanopore-based detection of DNA methylation

Onkar Kulkarni, Reuben Jacob Mathew, Rhea Jana, Lamuk Zaveri, Sreenivas Ara, Tulasi Nagabandi, Nitesh Kumar Singh, Karthik Bharadwaj Tallapaka, Divya Tej Sowpati

Supplementary Figure 1

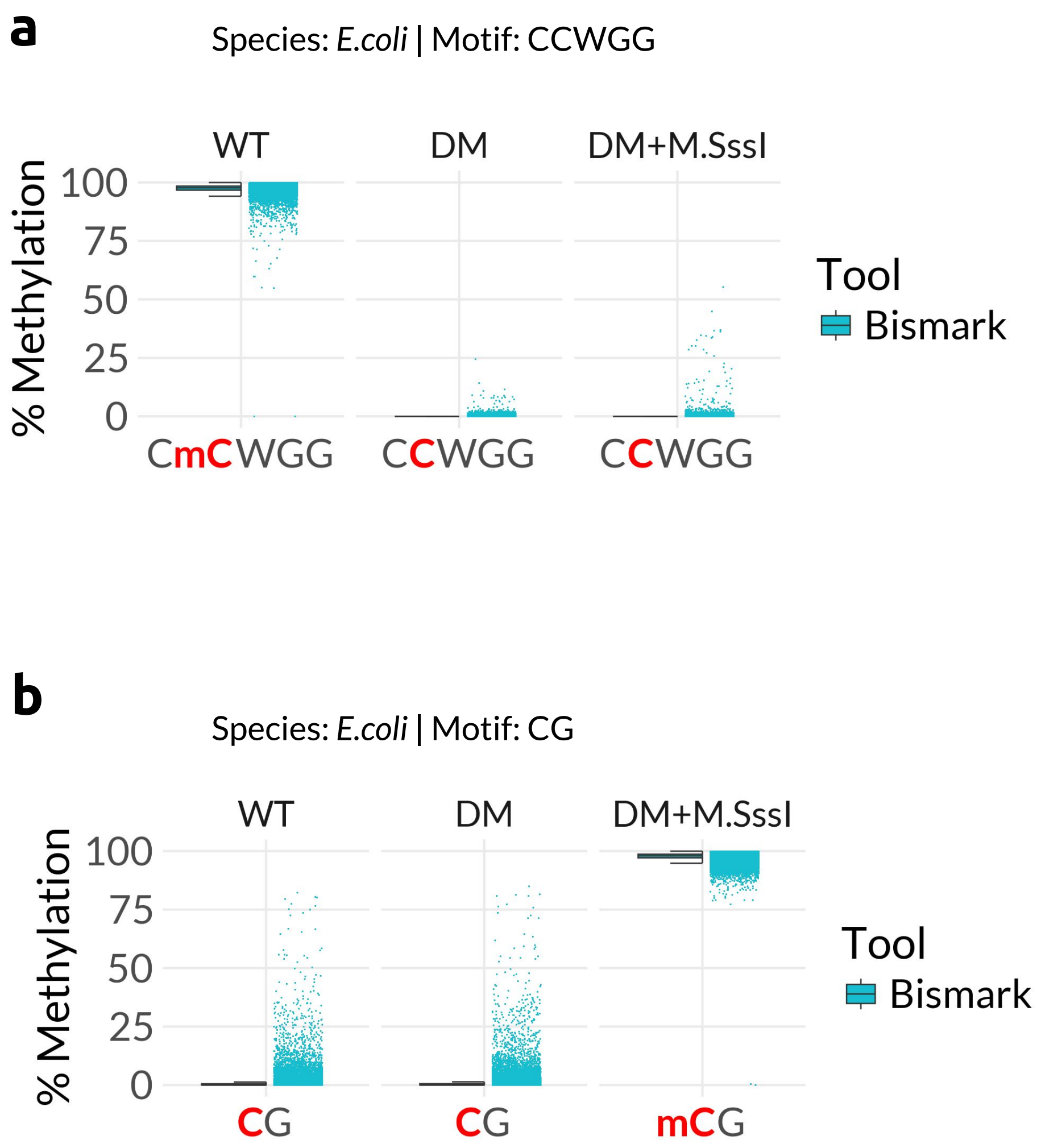

Supplementary Figure 1: Validation of different *E.coli* samples using EMSeq data (Bismark) visualized as Jitter-box plot. In Jitter-box plots, boxplot center lines indicate the median value, and lower and upper hinges represent the 25th and 75th percentiles, respectively. The whiskers denote 1.5x the interquartile range. The jitter part represents percent methylation for each site profiled as a dot. The color of the dot represents the tool used to generate that data. a) Jitter-box plot showing methylation percentage in CCWGG (n=24100) context for *E.coli* (WT, DM, DM+M.SssI) EMSeq data. b) Jitter-box plot showing methylation percentage in CpG (n=693586) context for *E.coli* (WT, DM, DM+M.SssI) EMSeq data.

Supplementary Figure 2

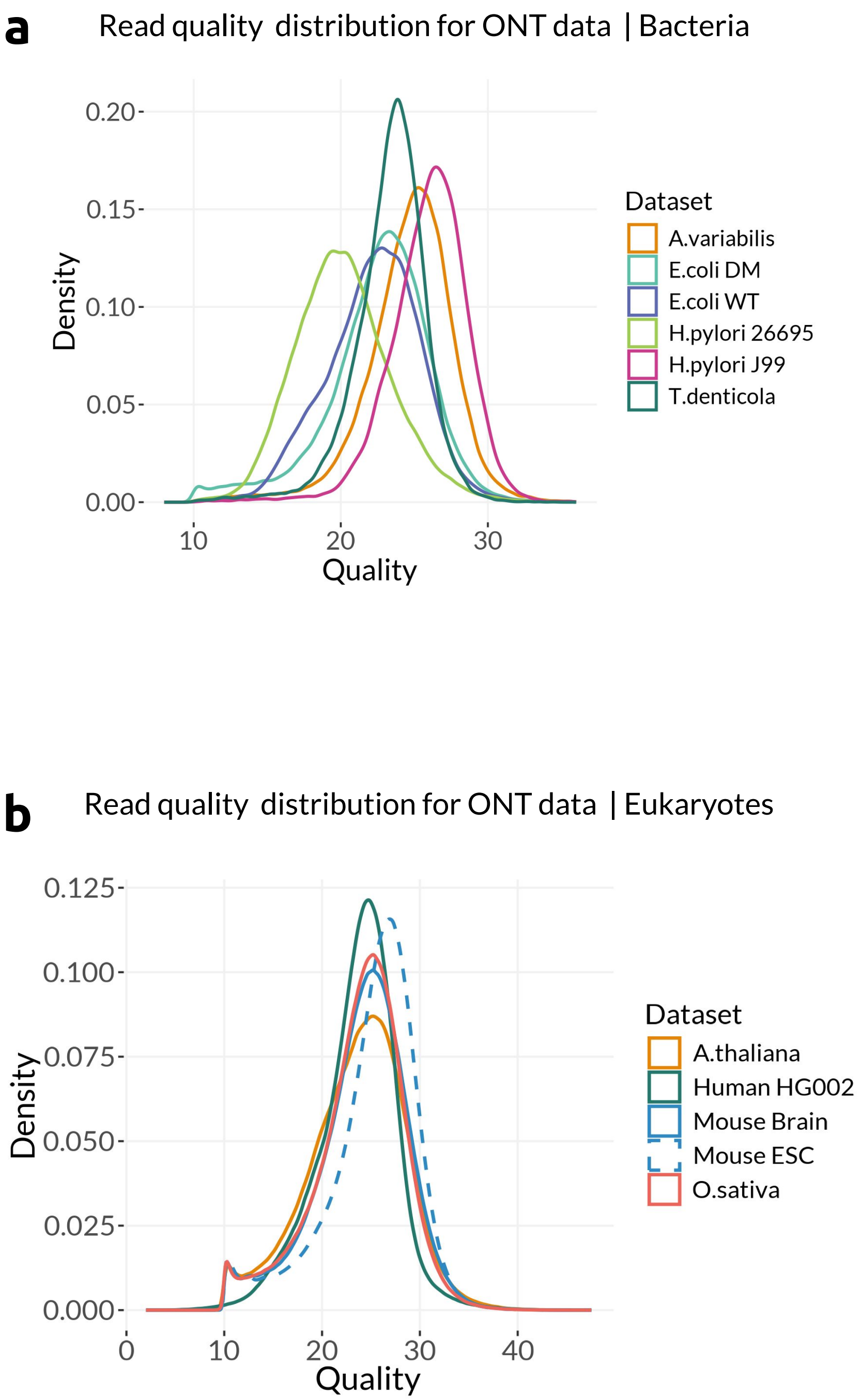

Supplementary Figure 2: Sequencing quality across various samples used for benchmarking. Density plots showing the variance of quality score distribution across ONT data for a) bacterial samples, and b) eukaryotic samples used in this study.

Supplementary Figure 3

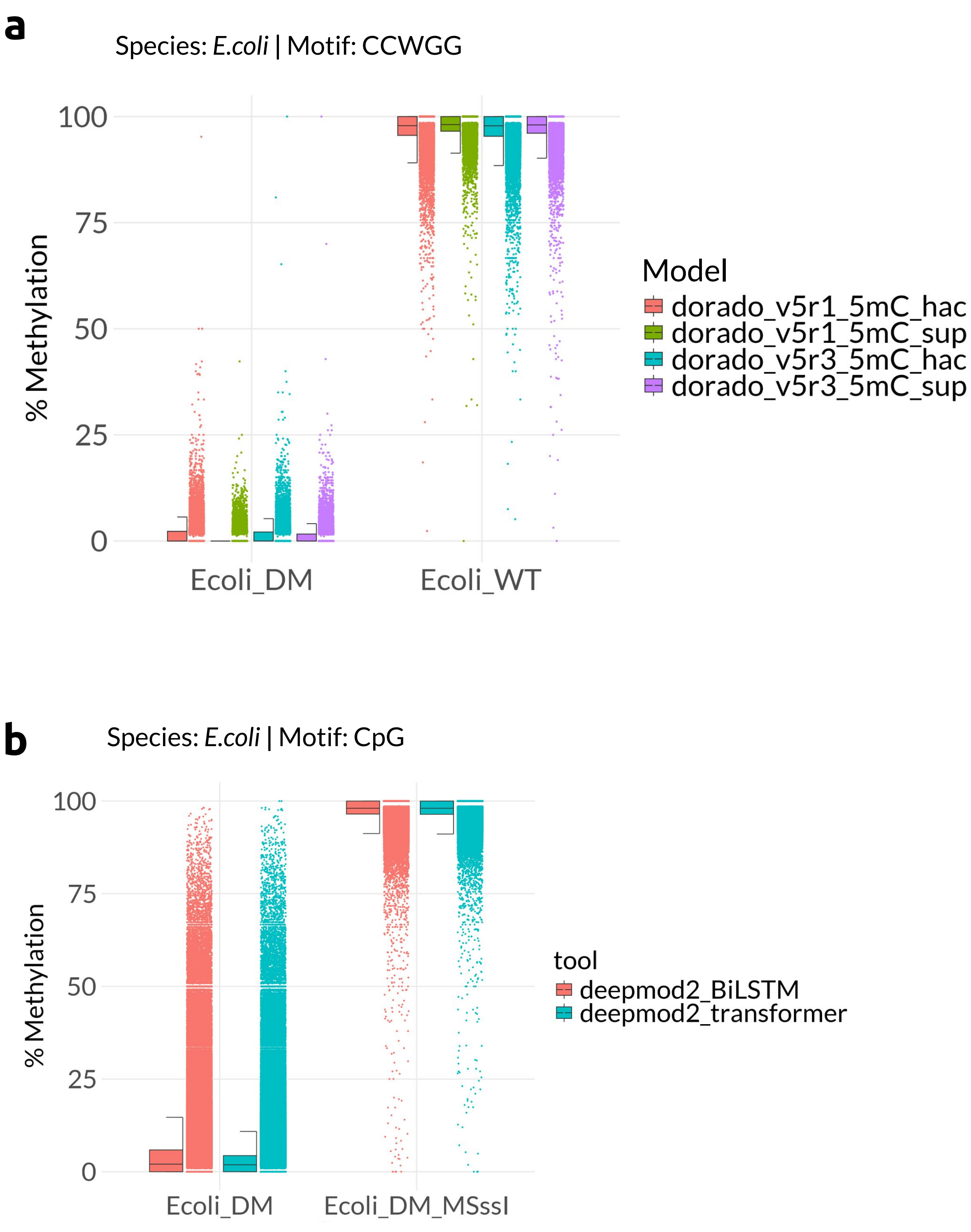

Supplementary Figure 3: Variance in performance of different model-subtypes offered by each tool on bacterial datasets. In Jitter-box plots, boxplot center lines indicate the median value, and lower and upper hinges represent the 25th and 75th percentiles, respectively. The whiskers denote 1.5x the interquartile range. a) Jitter-box plot depicting the CcWGG (n=24100) context performance differences between the high-accuracy (hac) and super-accuracy (sup) model variants offered by Dorado for *E.coli* WT and DM samples. b) Jitter-box plot depicting the CpG (n=693586) context performance differences between the BiLSTM and transformer model architectures offered by DeepMod2 in *E.coli* DM and DM+M.SssI samples.

Supplementary Figure 4

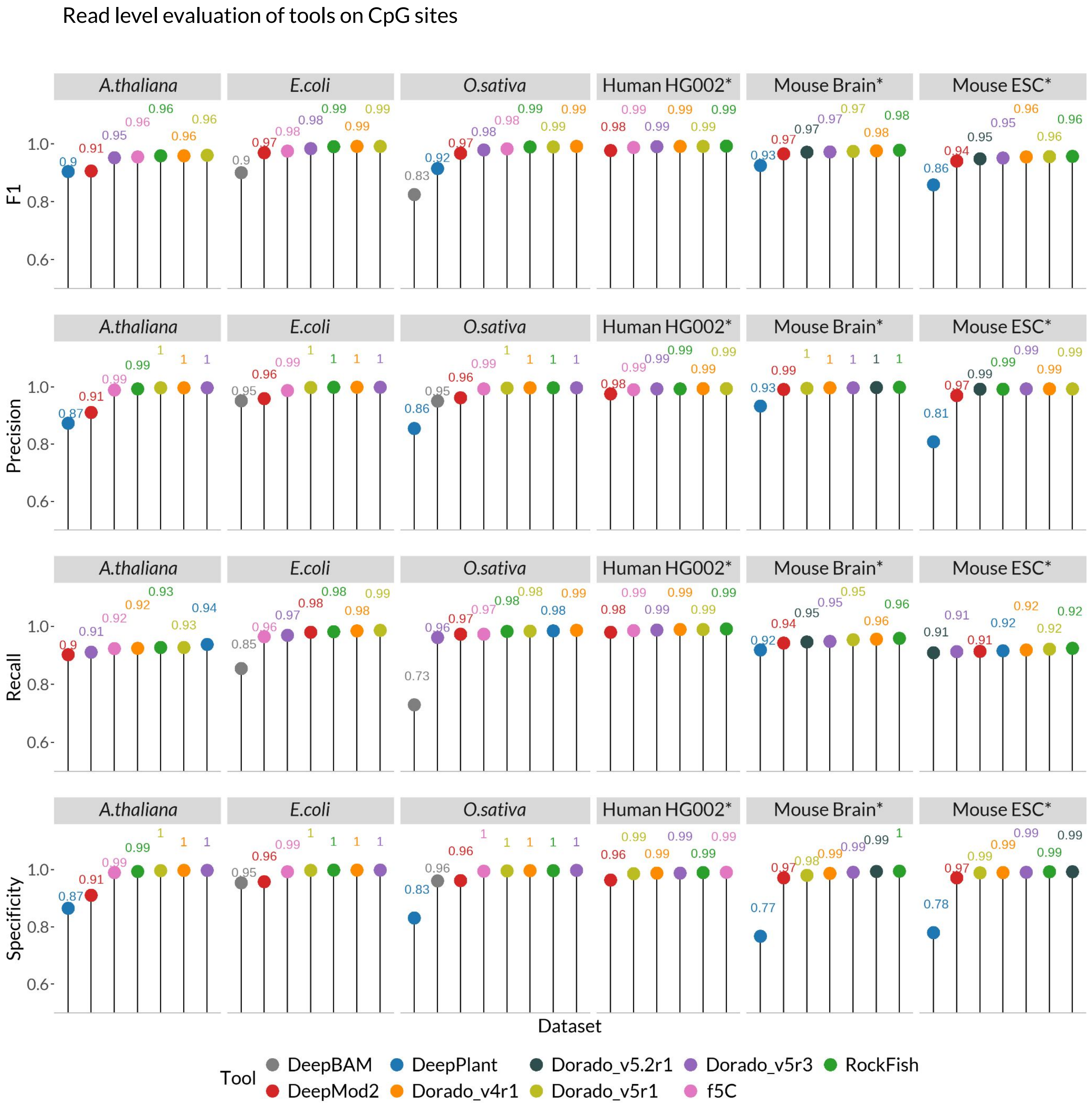

Supplementary Figure 4: Read level evaluation of various tools. Performance metrics of tools on CpG sites. F1, Precision, Recall, and Specificity are represented as lollipops colored by tools for each dataset. The score is also indicated next to each lollipop for clarity. The asterisk next to Human HG002, mouse brain and ESC indicates that the calculations are done using data of only chromosome 1. The y-axis is scaled from 0.5 instead of 0 to highlight subtle differences in the scores.

Supplementary Figure 5

Effect of probability thresholds on DeepPlant's performance

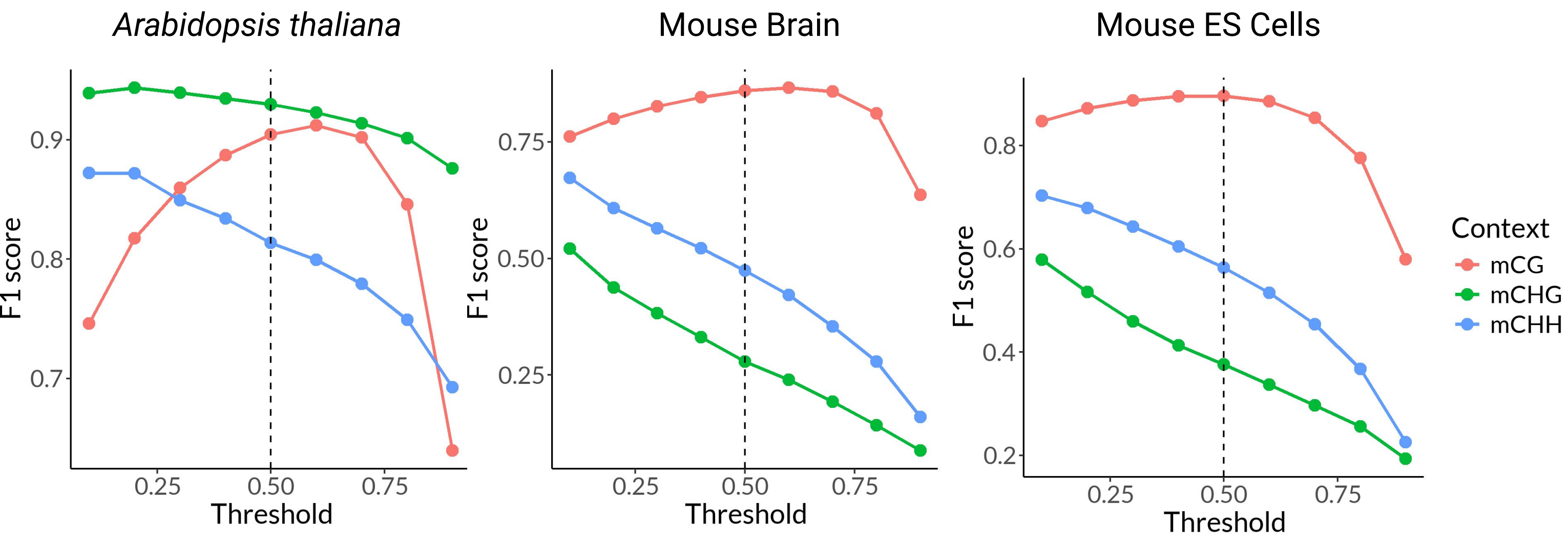

Supplementary Figure 5: Read-level evaluation of the effect of probability thresholds on DeepPlant's performance. The F1 scores (y-axis) are plotted in a line plot against the thresholds (x-axis) of DeepPlant for CG, CHG, and CHH contexts of *A.thaliana*, mouse brain and ES cells. The vertical dashed line indicates the default threshold (0.5) used by DeepPlant.

Supplementary Figure 6

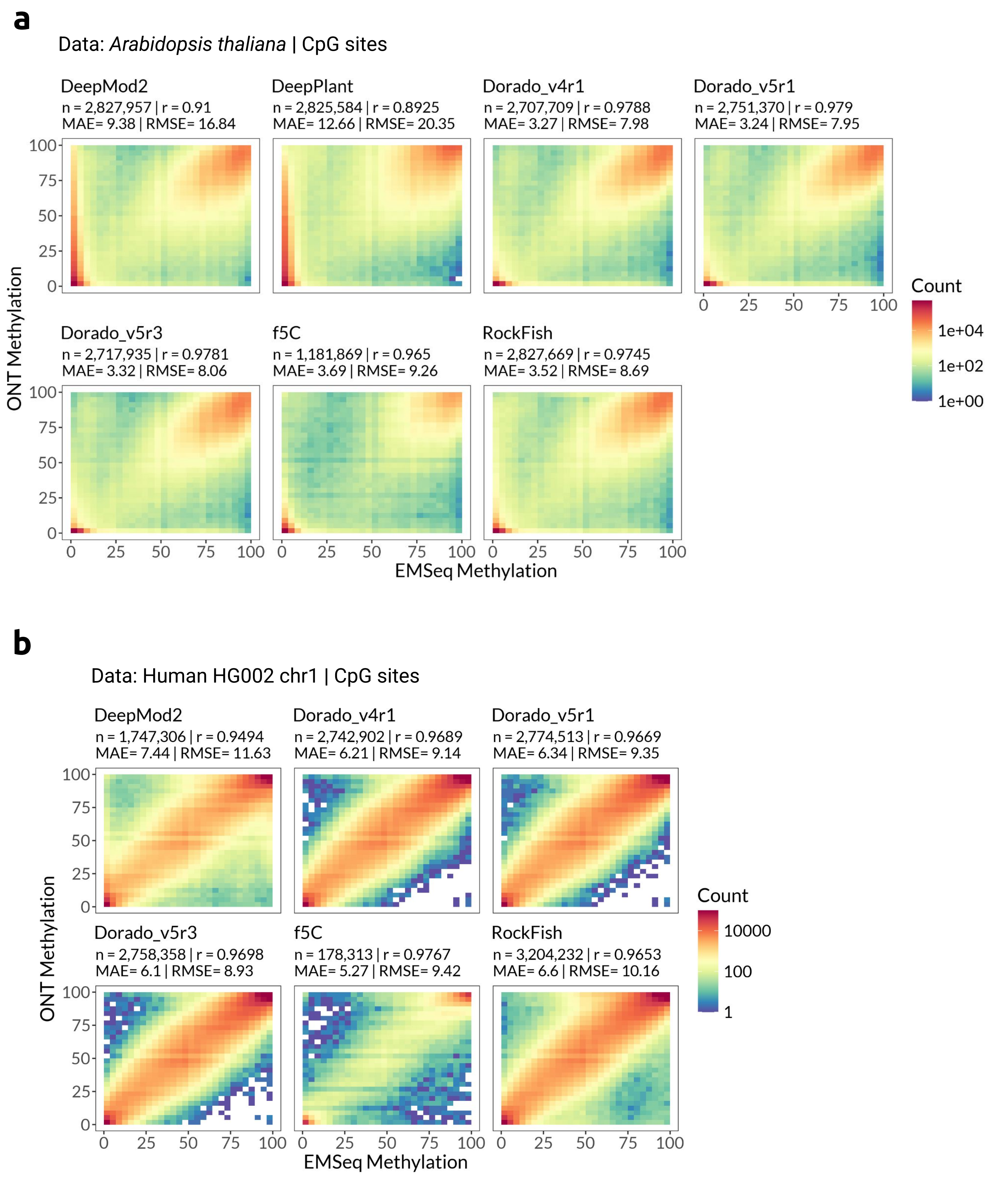

Supplementary Figure 6: Site-level evaluation of methylation calling tools using correlation heatmaps at CpG sites for Arabidopsis and Human data. n = number of CpG sites profiled and covered by >=20 reads; r = Pearson correlation coefficient; MAE = Mean Absolute Error; RMSE = Root Mean-Squared Error. a-b) Correlation heatmaps of CpG sites from *A.thaliana* data (a) and human chromosome 1 of HG002 data (b), for each tool compared to EMSeq data (ground truth).

Supplementary Figure 7

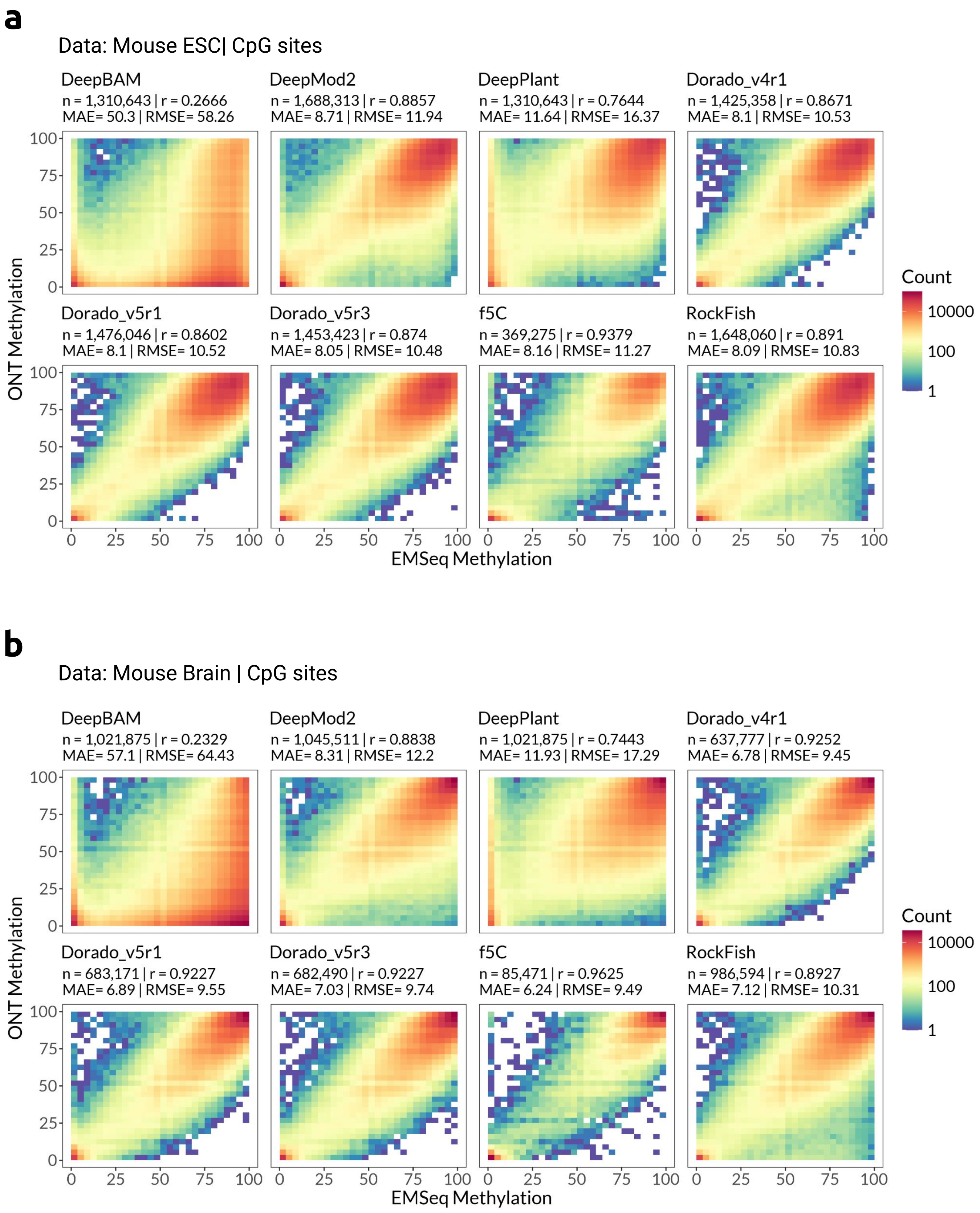

Supplementary Figure 7: Site-level evaluation of methylation calling tools using correlation heatmaps in CpG context for mouse brain and ESC data. n = number of CpG sites profiled and covered by >=20 reads; r = Pearson correlation coefficient; MAE = Mean Absolute Error; RMSE = Root Mean-Squared Error. a-b) Correlation heatmaps of CpG sites from mouse embryonic stem cells (a) and mouse brain (b) data, for each tool compared to EMSeq data (ground truth)

Supplementary Figure 8

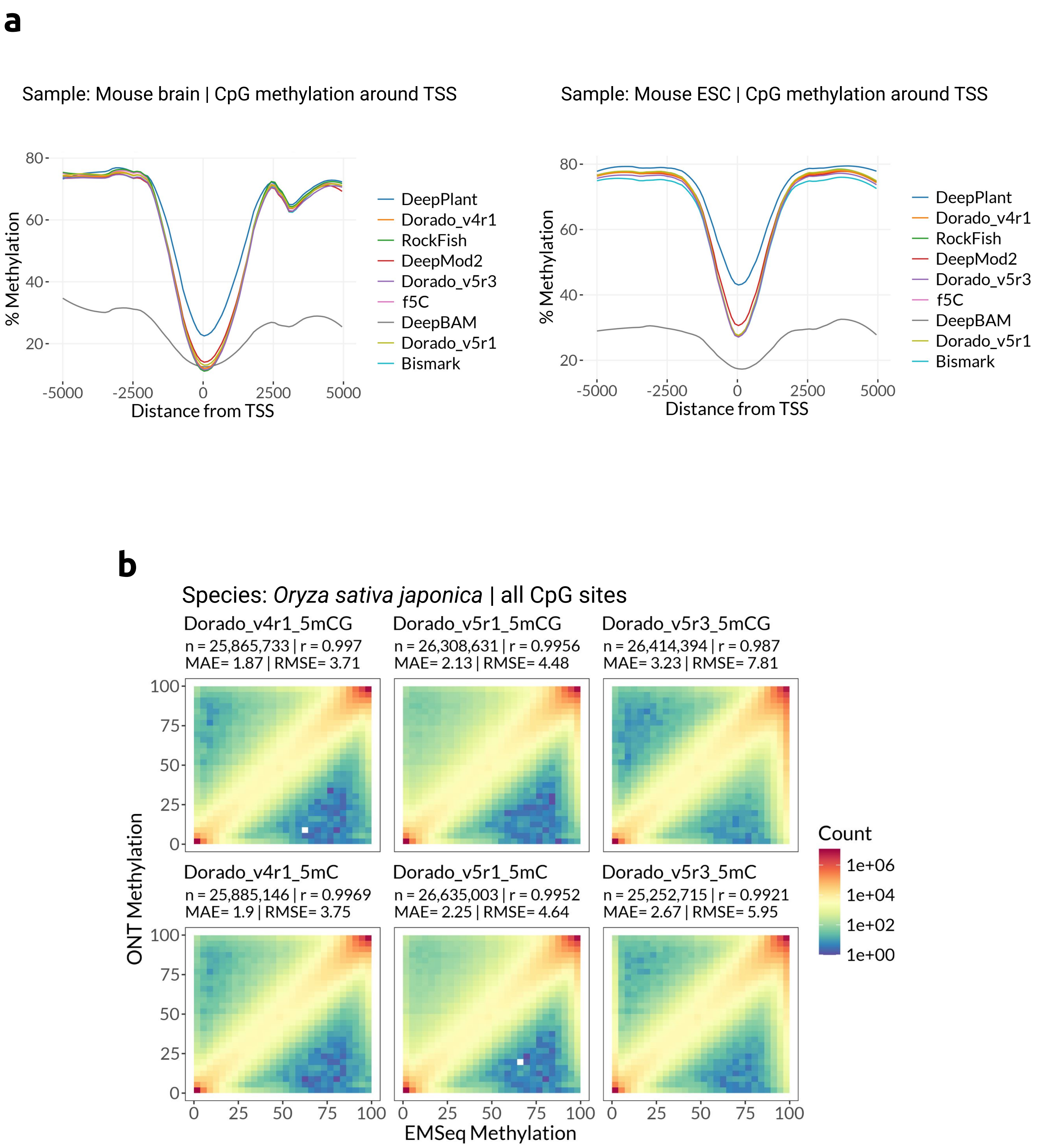

Supplementary Figure 8: CpG methylation calling around Transcription Start Sites (mouse) and correlation heatmaps on rice data. a) The methylation percentage of CpG sites spanning 5kb upstream and downstream of the transcription start sites (TSS) on chromosome 1 plotted as a line plot for each methylation tool, for mouse brain and ESC data. b) Evaluation of CpG-sites from rice data. n = number of CpG sites profiled and covered by >=20 reads; r = Pearson correlation coefficient; MAE = Mean Absolute Error; RMSE = Root Mean-Squared Error. Correlation heatmap of all CpG sites profiled by each of the Dorado all-context 5mC and 5mCG models compared to the EMSeq ground truth.

Supplementary Figure 9

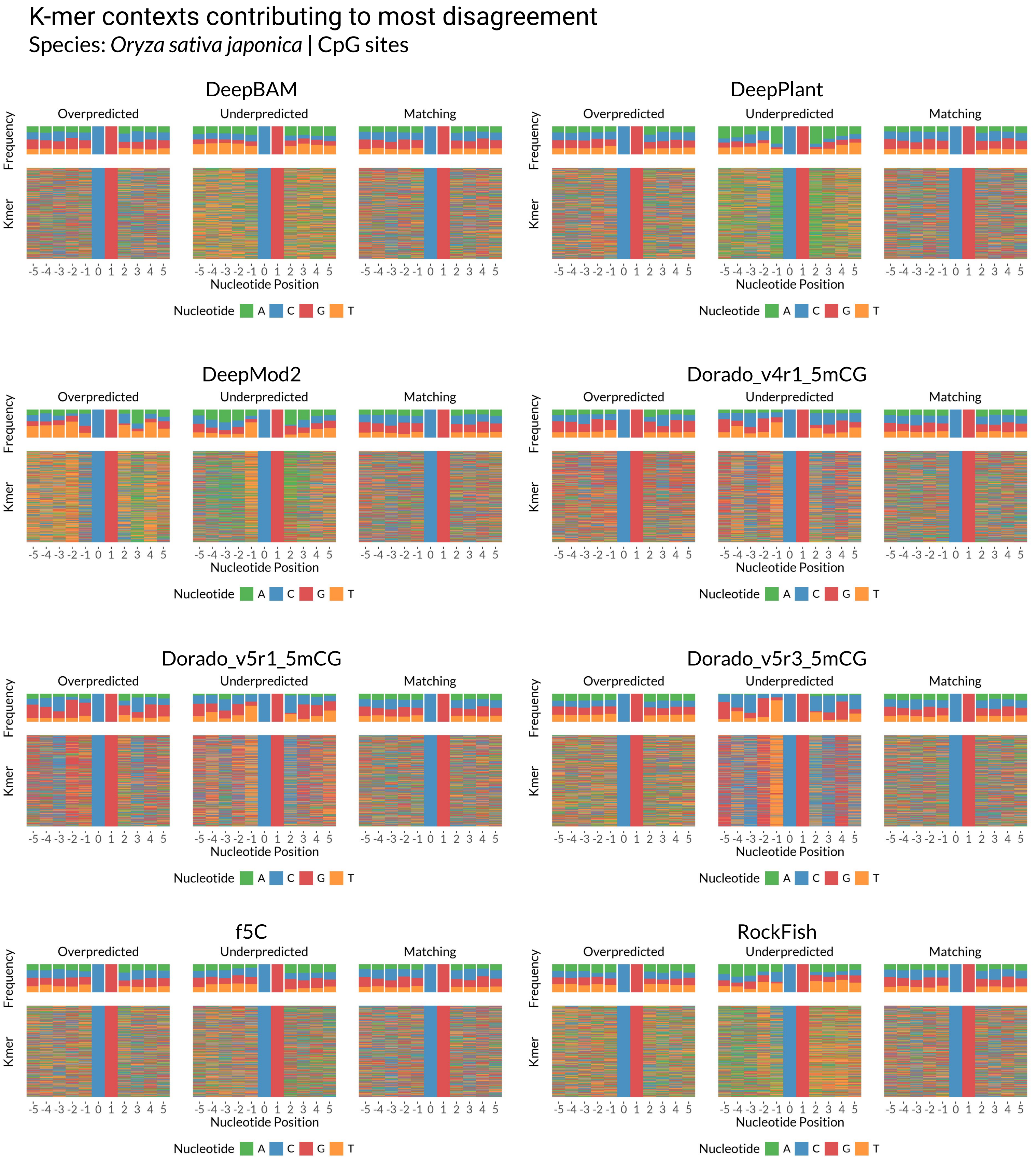

Supplementary Figure 9: K-mers of disagreement at CpG locations on rice data. 5000 most disagreeing locations were plotted in each case; Overpredicted - the nanopore tool calls more methylation than ground-truth, Underpredicted - nanopore tool calls less methylation compared to ground-truth. Matching - 5000 randomly sampled locations where the difference of methylation level between the ground truth and reported value is 0.

Supplementary Figure 10

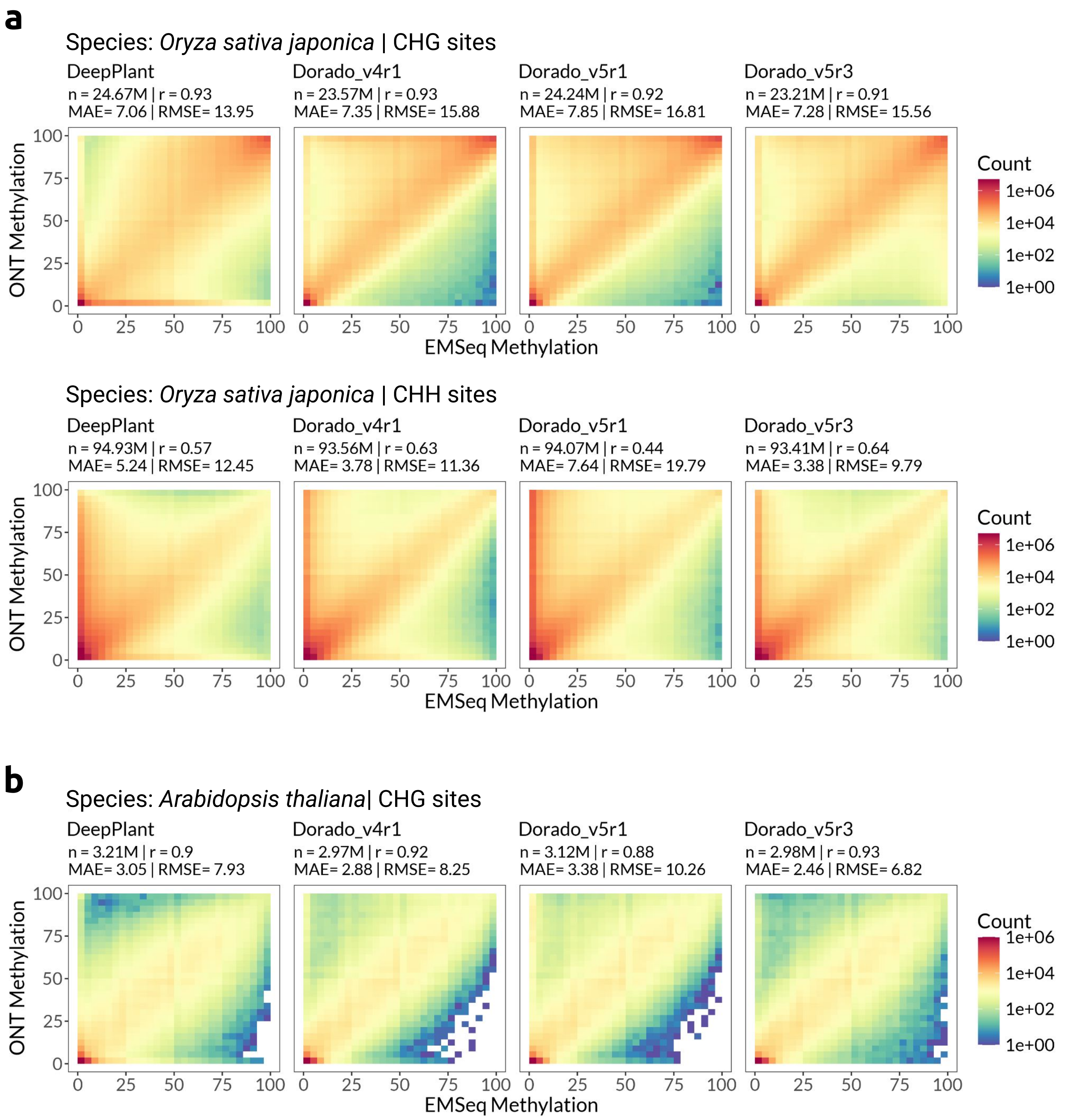

Supplementary Figure 10: Site-level evaluation of methylation calling tools using correlation heatmaps in non-CG context, on plant data. n = number of sites profiled and covered by >=20 reads; r = Pearson correlation coefficient; MAE = Mean Absolute Error; RMSE = Root Mean-Squared Error. a) Correlation heatmap of CHG (top) and CHH (bottom) sites from *O.sativa* data for each tool compared to the EMSeq ground truth. b) Correlation heatmap of CHG sites from *A.thaliana* data for each tool compared to the EMSeq data ground truth.

Supplementary Figure 11

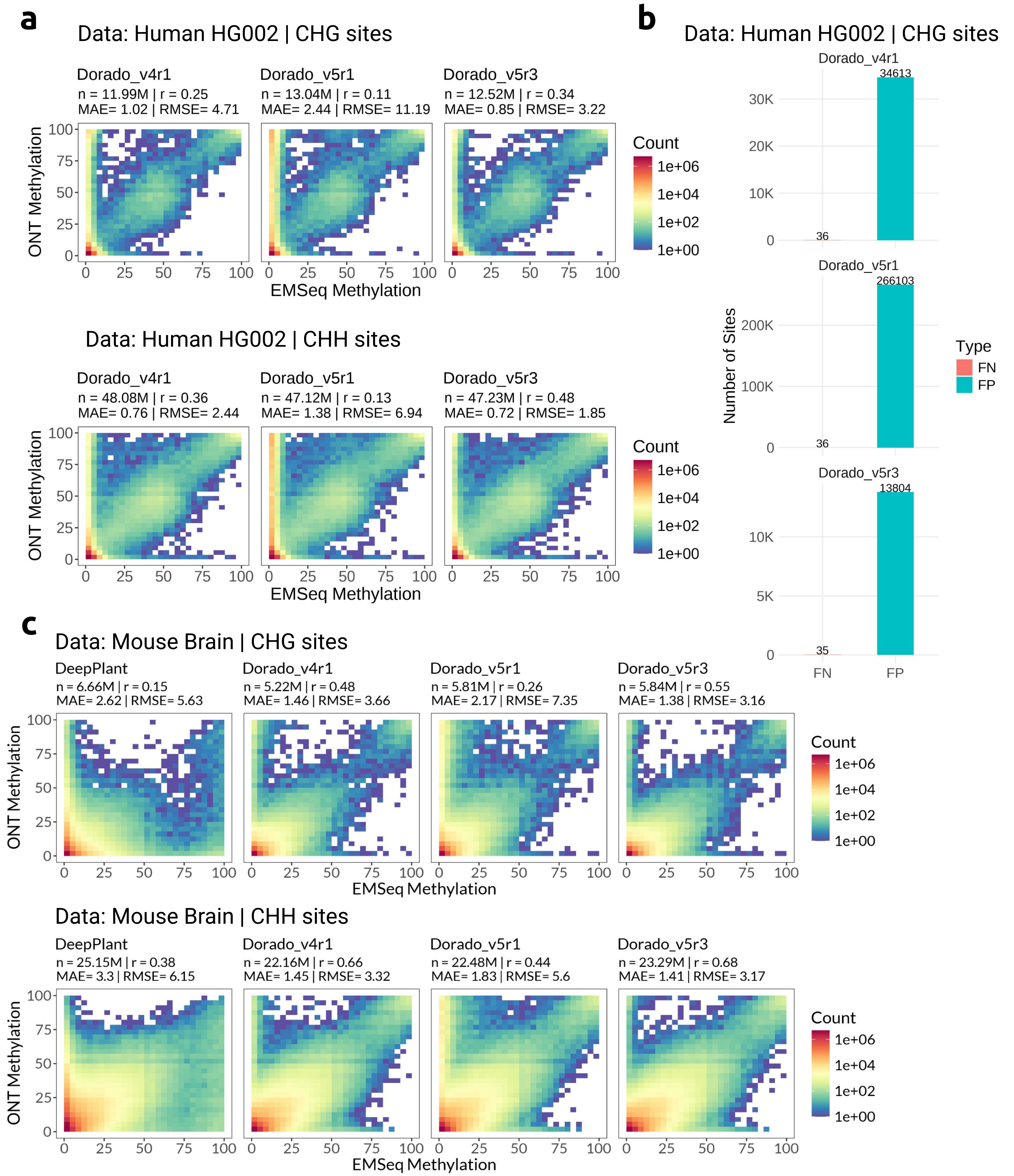

Supplementary Figure 11: Site-level evaluation of non-CG methylation using correlation heatmaps, on human HG002 and mouse brain data. n = number of sites profiled and covered by >=20 reads; r = Pearson correlation coefficient; MAE = Mean Absolute Error; RMSE = Root Mean-Squared Error. a) Correlation heatmap of CHG (top) and CHH (bottom) sites from chromosome 1 of human data for the Dorado all-context 5mC models (a), Number of locations reported to be <5% methylated by EMSeq and >25% by nanopore models (representing FPs) and vice versa; <5% by nanopore models and >25% by EMSeq (representing FNs) for Dorado models in CHG context, using the HG002 dataset (b), chr 1 of mouse brain data for DeepPlant and Dorado all-context 5mC models (b) compared to the EMSeq ground truth.

Supplementary Figure 12

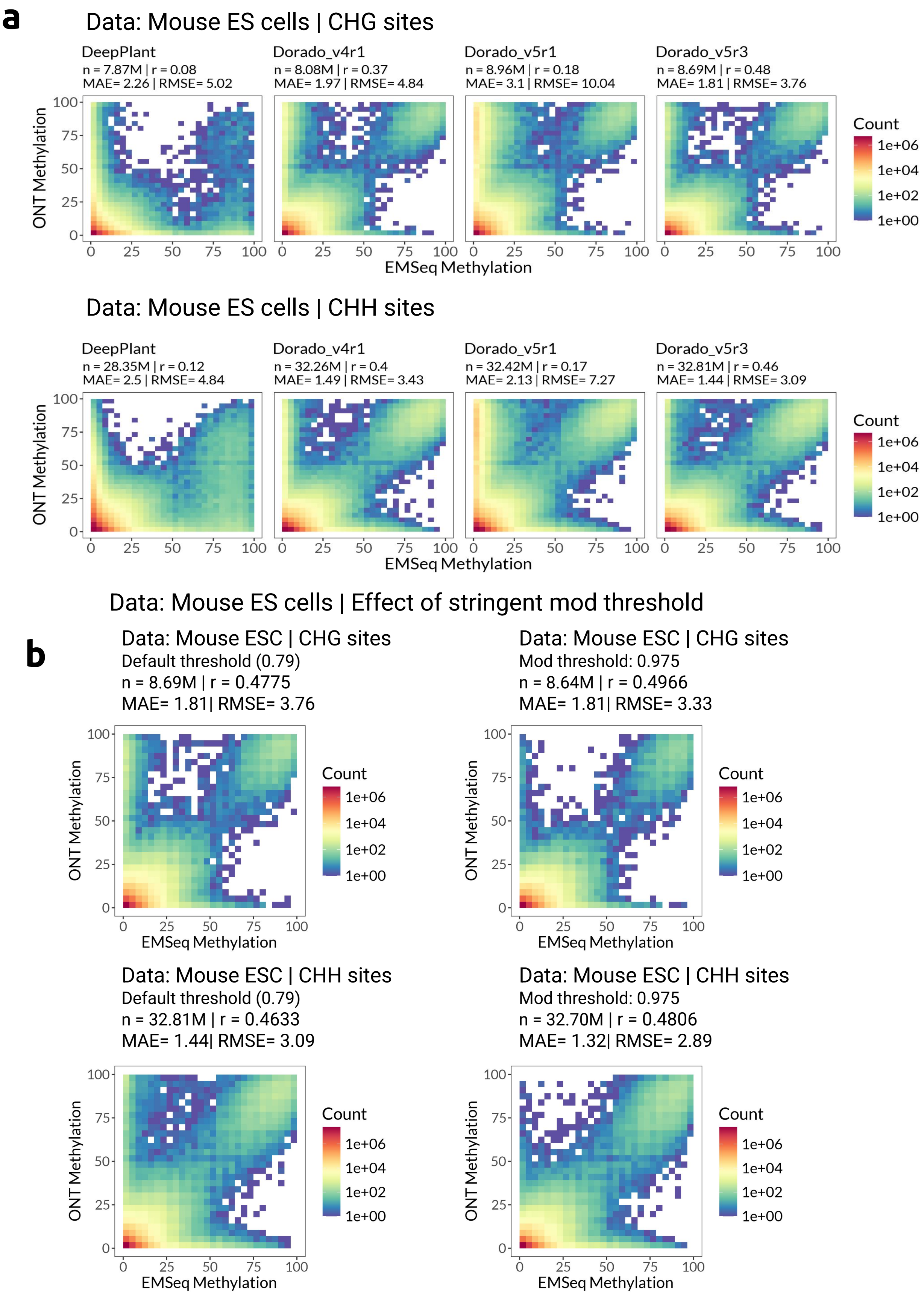

Supplementary Figure 12: Site-level evaluation of non-CG methylation using correlation heatmaps on mouse ESC data, and effect of adjusting probability thresholds on false positives. n = number of sites profiled and covered by >=20 reads; r = Pearson correlation coefficient; MAE = Mean Absolute Error; RMSE = Root Mean-Squared Error. a-b) Correlation heatmap of CHG (top) and CHH (bottom) sites from mouse ESC chromosome 1 data for (a) each tool compared to the EMSeq ground truth. (b) Dorado v5r3 model with default threshold (0.79, left) and a stringent mod threshold (0.97, right) compared with the EMseq ground truth.

Supplementary Figure 13

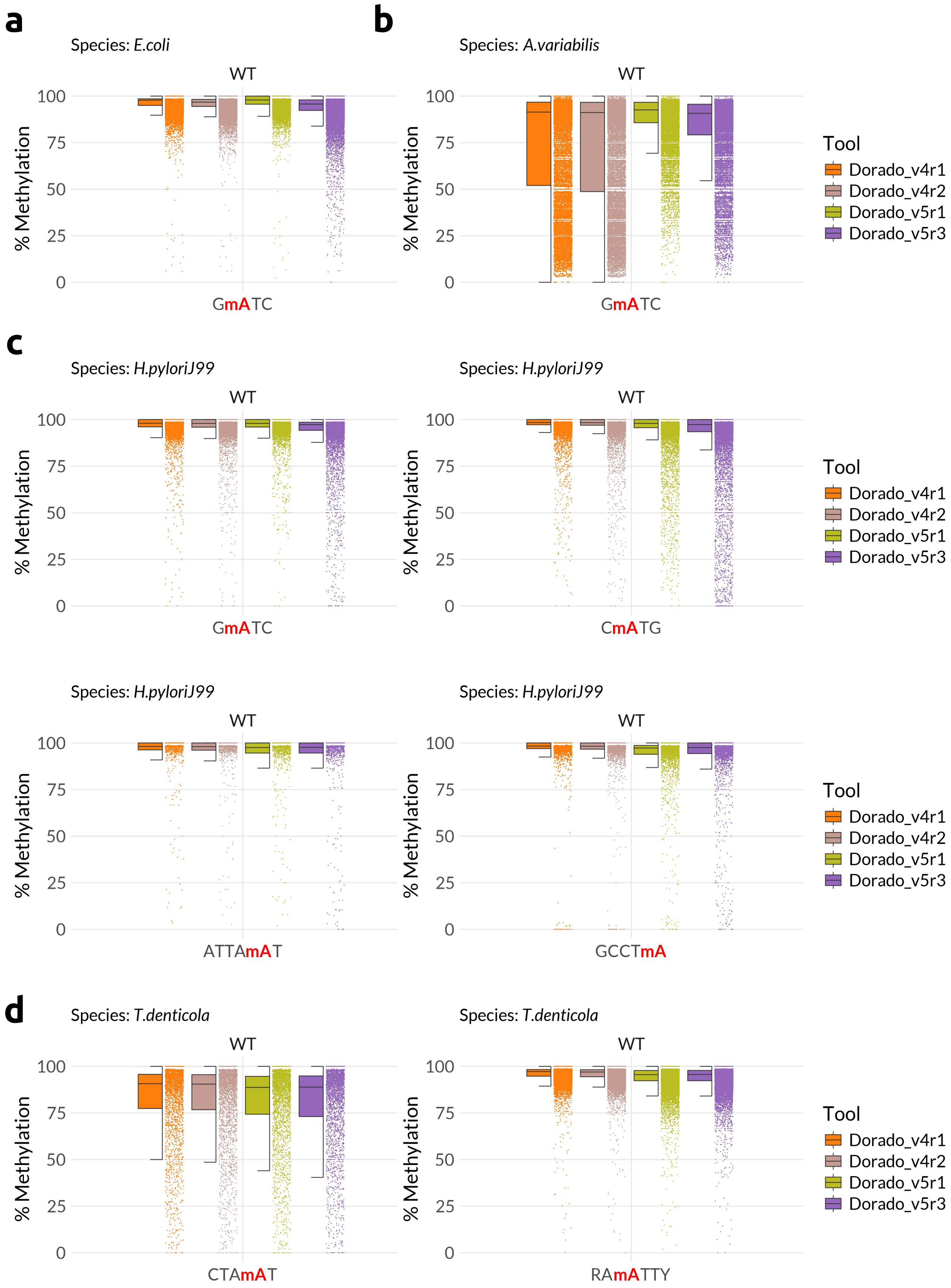

Supplementary Figure 13: Context-wise evaluation of 6mA calling tools. In Jitter-box plots, boxplot center lines indicate the median value, and lower and upper hinges represent the 25th and 75th percentiles, respectively. The whiskers denote 1.5x the interquartile range. a-d) Jitter-box plots depicting each tool's performance on GATC (n=38248) context in *E.coli* WT sample (a), GATC (n=34026) context in *A.variabilis* WT sample (b), GATC (n=11140), CATG (n=15336), ATTAAT (n=862), GCCTA (n=3214) contexts in *H.pylori* J99 WT sample (c) and CTAAT (n=2984), RAATTY (n=20044) contexts in *T.denticola* WT sample (d).

Supplementary Figure 14

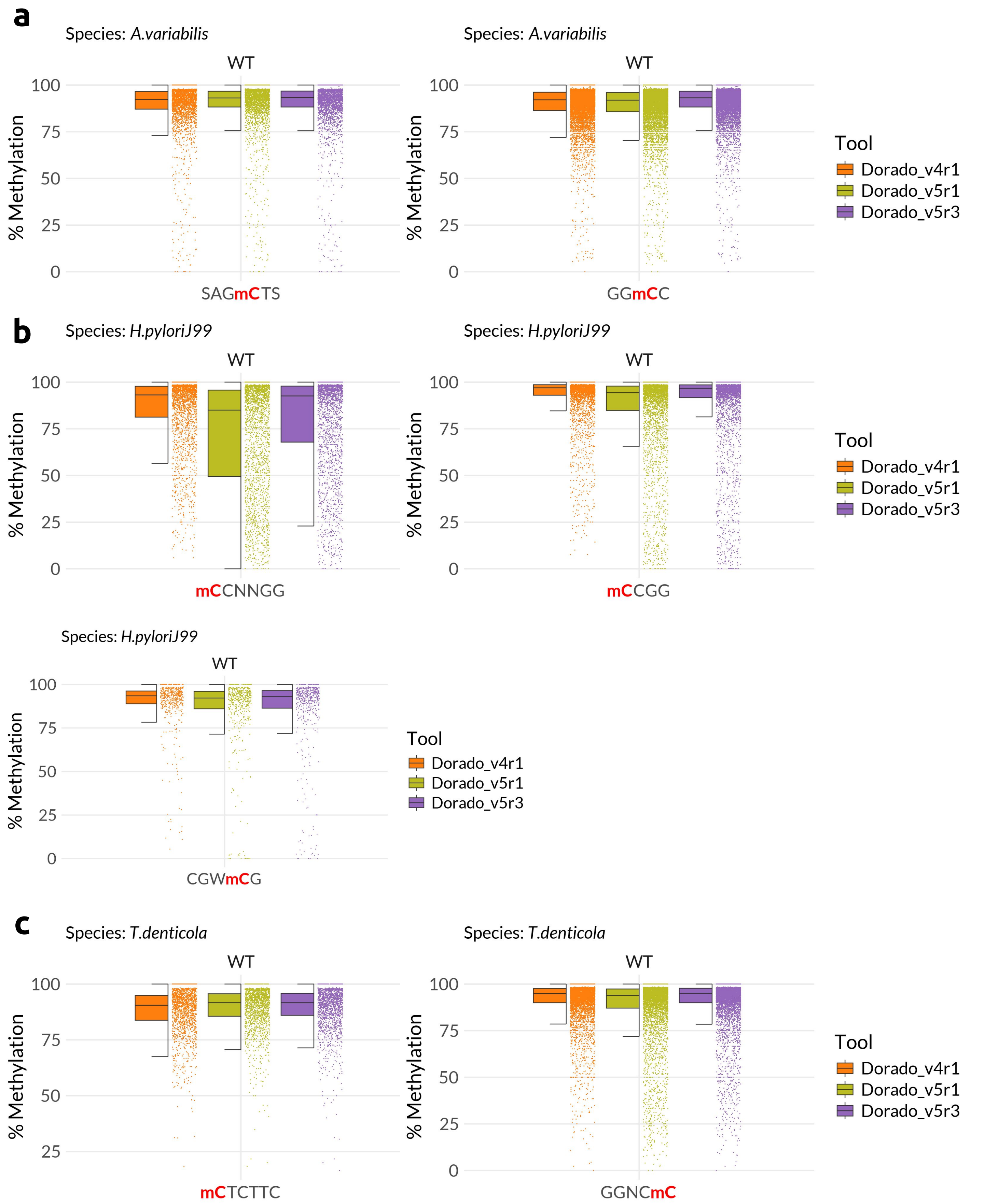

Supplementary Figure 14: Context-wise evaluation of 4mC calling tools. In Jitter-box plots, boxplot center lines indicate the median value, and lower and upper hinges represent the 25th and 75th percentiles, respectively. The whiskers denote 1.5x the interquartile range. a-c) Jitter-box plots depicting each tool's performance on SAGCTS (n=2942), GGCC (n=10050) contexts in *A.variabilis* WT sample (a), CCNNGG (n=2408), CCGG (n=3630), CGWCG (n=534) contexts in *H.pylori* J99 WT sample (b) and CTCTTC (n=1695), GGNCC (n=5844) contexts in *T.denticola* WT sample (c).

Supplementary Figure 15

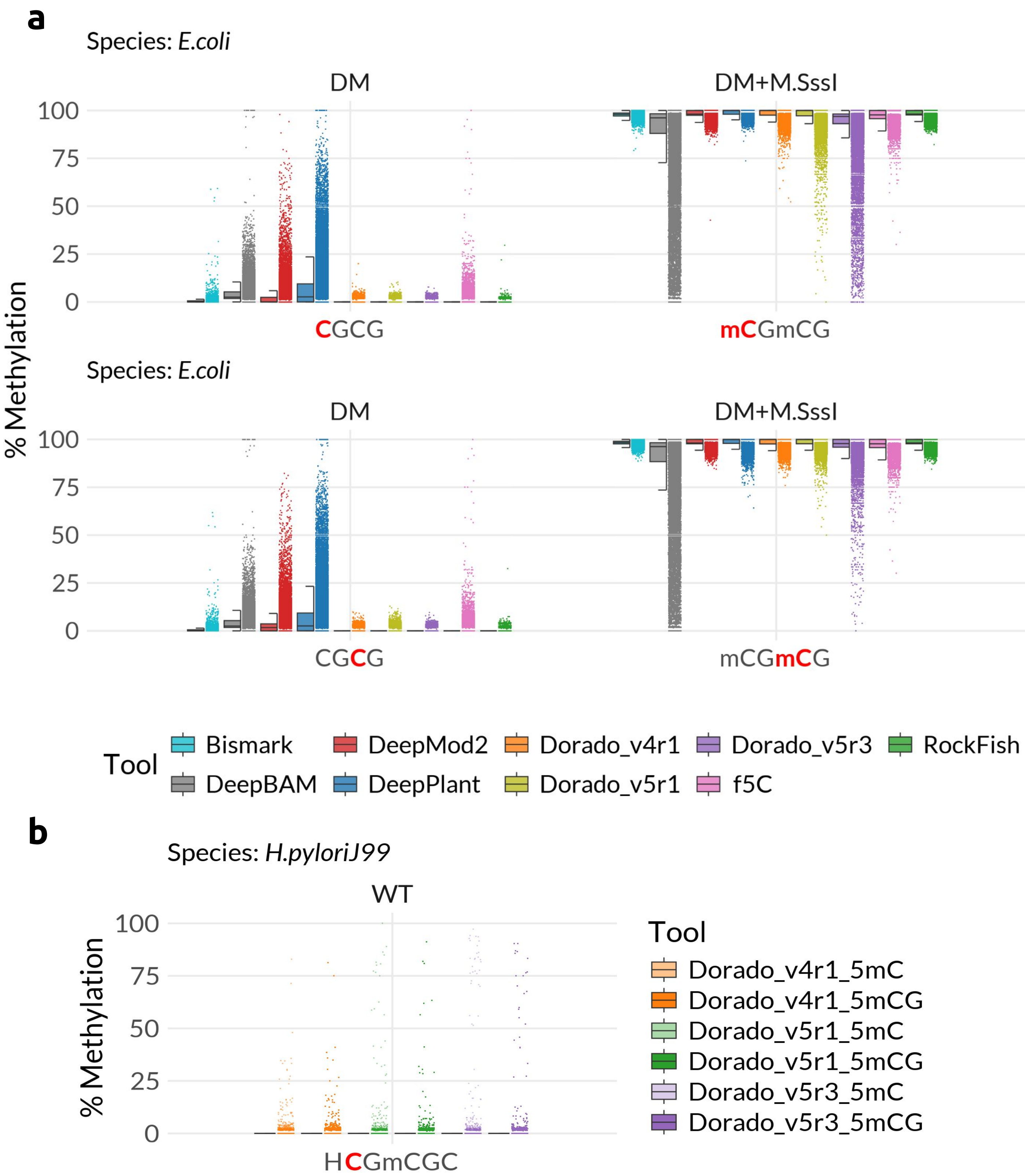

Supplementary Figure 15: Evaluating the effect of neighboring modifications for 5mCG calling tools. The profiled C is shown in red and the methylation status is indicated by a preceding "m". In Jitter-box plots, boxplot center lines indicate the median value, and lower and upper hinges represent the 25th and 75th percentiles, respectively. The whiskers denote 1.5x the interquartile range. a) Jitter-box plot depicting each tool's performance on the CGCG (n=56452) context in *E.coli* DM and DM+M.SssI samples. Both the consecutive CGs are expected to be methylated in the latter sample. b) Jitter-box plot depicting the performance of Dorado all-context 5mC and 5mCG models on HCGmCGC (n=2225) context in *H.pylori* J99 WT sample. The profiled C, depicted in red is part of a CG motif preceding an mCG.

Supplementary Figure 16

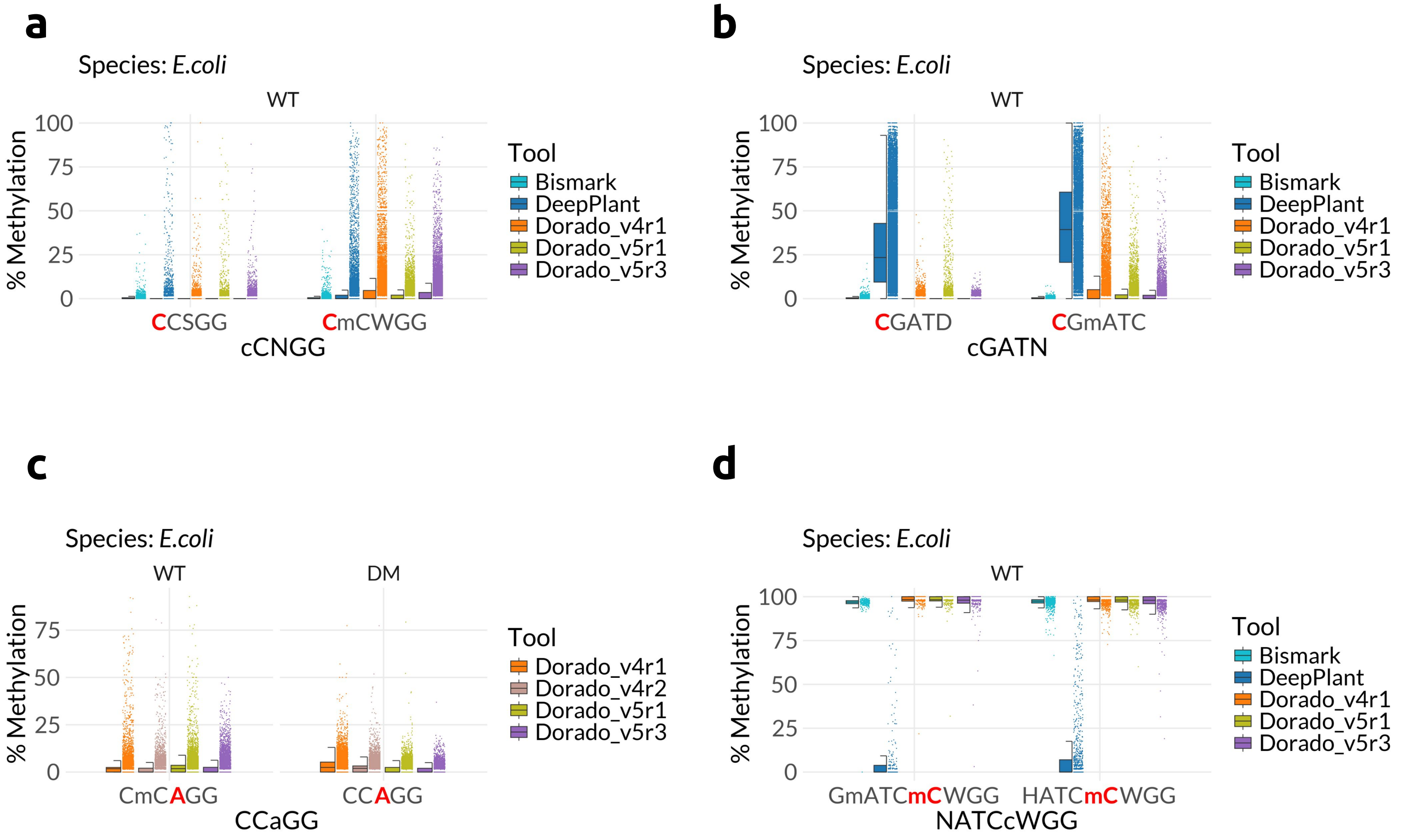

Supplementary Figure 16: Evaluating the effect of neighboring modifications in various contexts. Profiled base is shown in red. The methylation status of the bases is indicated with a preceding “m”. In Jitter-box plots, boxplot center lines indicate the median value, and lower and upper hinges represent the 25th and 75th percentiles, respectively. The whiskers denote 1.5x the interquartile range. a) Jitter-box plot depicting effect of mC on umC for each tool in CCNGG context (CCSGG, n=18810, unmethylated; CCWGG, n=24100, methylated) in *E.coli* WT sample. b) Jitter-box plot depicting effect of mC on umC for each tool in cGATN context (CGATD, n=37666, unmethylated; CGATC, n=10956, methylated) in *E.coli* WT sample. c) Jitter-box plot depicting effect of mC on umA for each tool in CCAGG (n=12050) context in *E.coli* DM (unmethylated) and WT (methylated) samples. d) Jitter-box plot depicting effect of mA on mC for each tool in NATCCWGG context (HATCCWGG, n=1099, unmethylated; GATCCWGG, n=264, methylated) in *E.coli* WT sample.

Supplementary Figure 17

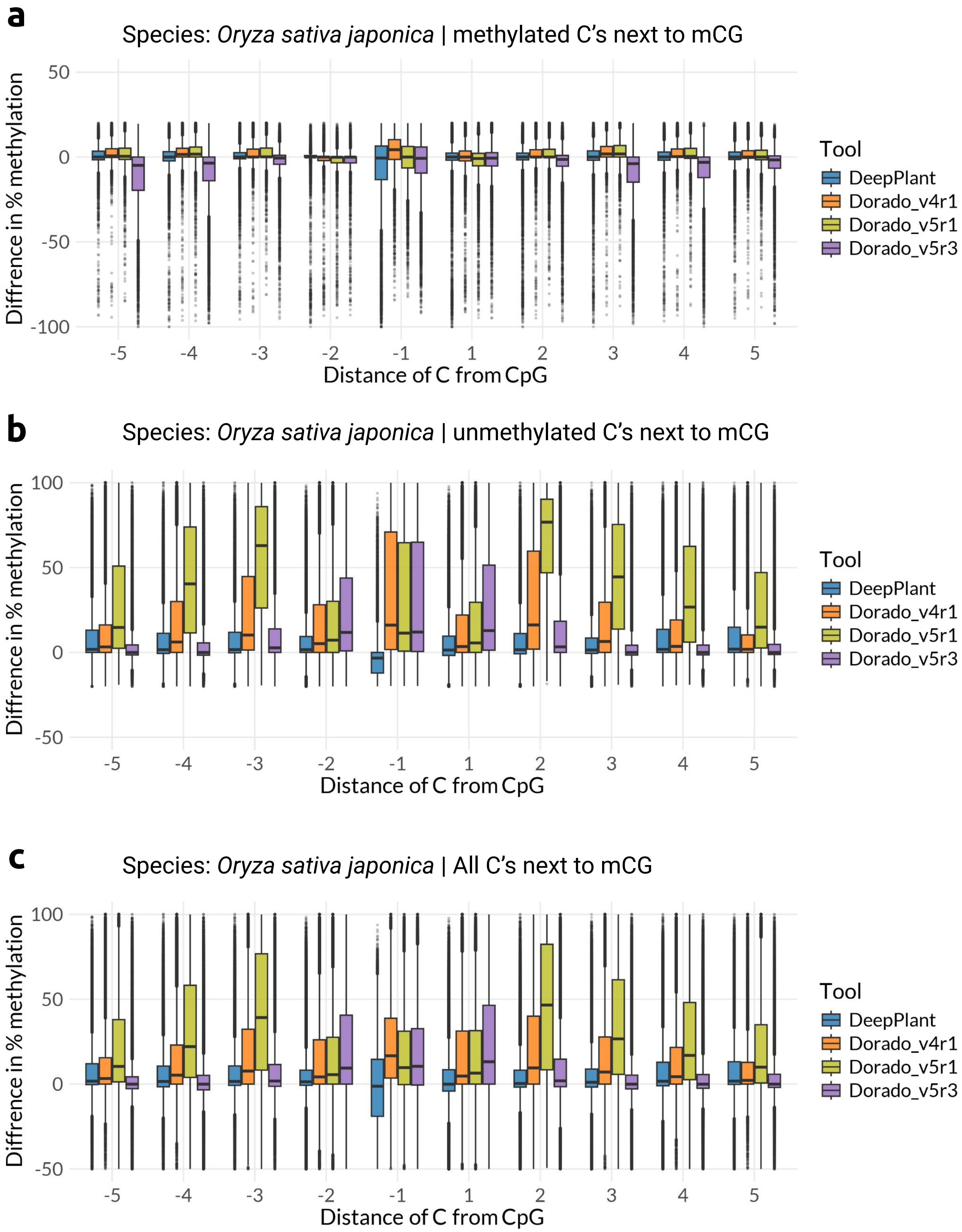

Supplementary Figure 17: Evaluating the effect of neighboring CG methylation on rice data. The difference in methylation calls for a cytosine as a function of its distance from methylated CpG. Negative values indicate underestimation of methylation near mCG. EMSeq data used as ground truth. Boxplot center lines indicate the median value, and lower and upper hinges represent the 25th and 75th percentiles, respectively. The whiskers denote 1.5x the interquartile range. a-c) Boxplot showing difference in methylation calls from the tool and EMSeq data for sites where profiled C is methylated (>=80% methylation) (a), profiled C is unmethylated (<=20% methylation) (b) and for all Cs neighboring mCG (c) in *O.sativa*.

Supplementary Figure 18

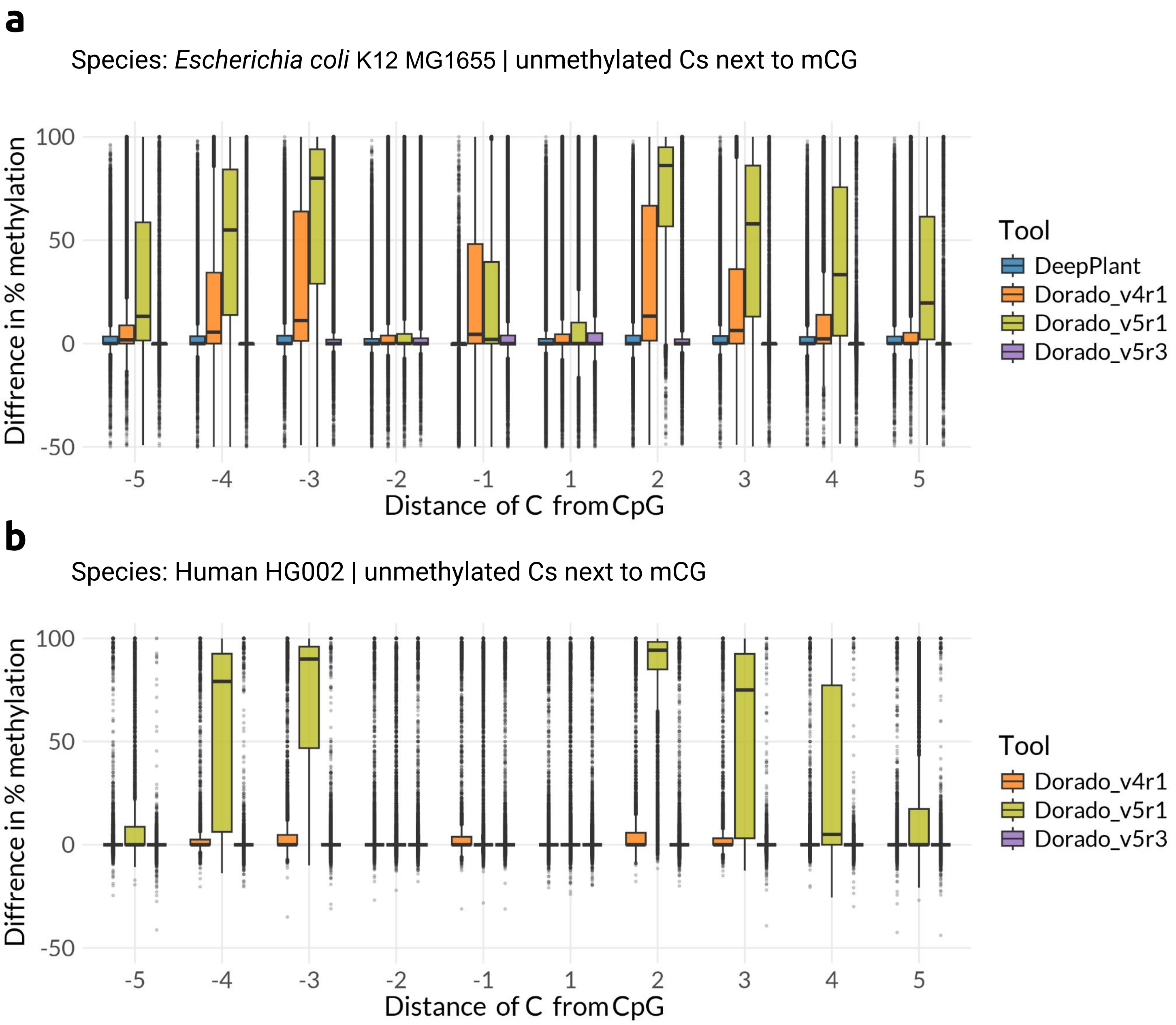

Supplementary Figure 18: Evaluating the effect of neighboring CG methylation on *E.coli* and human data. The difference in methylation calls for a cytosine as a function of its distance from methylated CpG. Negative values indicate underestimation of methylation near mCG. EMSeq data is used as ground truth. Boxplot center lines indicate the median value, and lower and upper hinges represent the 25th and 75th percentiles, respectively. The whiskers denote 1.5x the interquartile range. a-b) Boxplots showing difference in methylation calls from the tool and EMSeq data for unmethylated Cs neighboring mCG in *E.coli* (a), unmethylated Cs neighboring mCG in human data (b).

Supplementary Figure 19

a

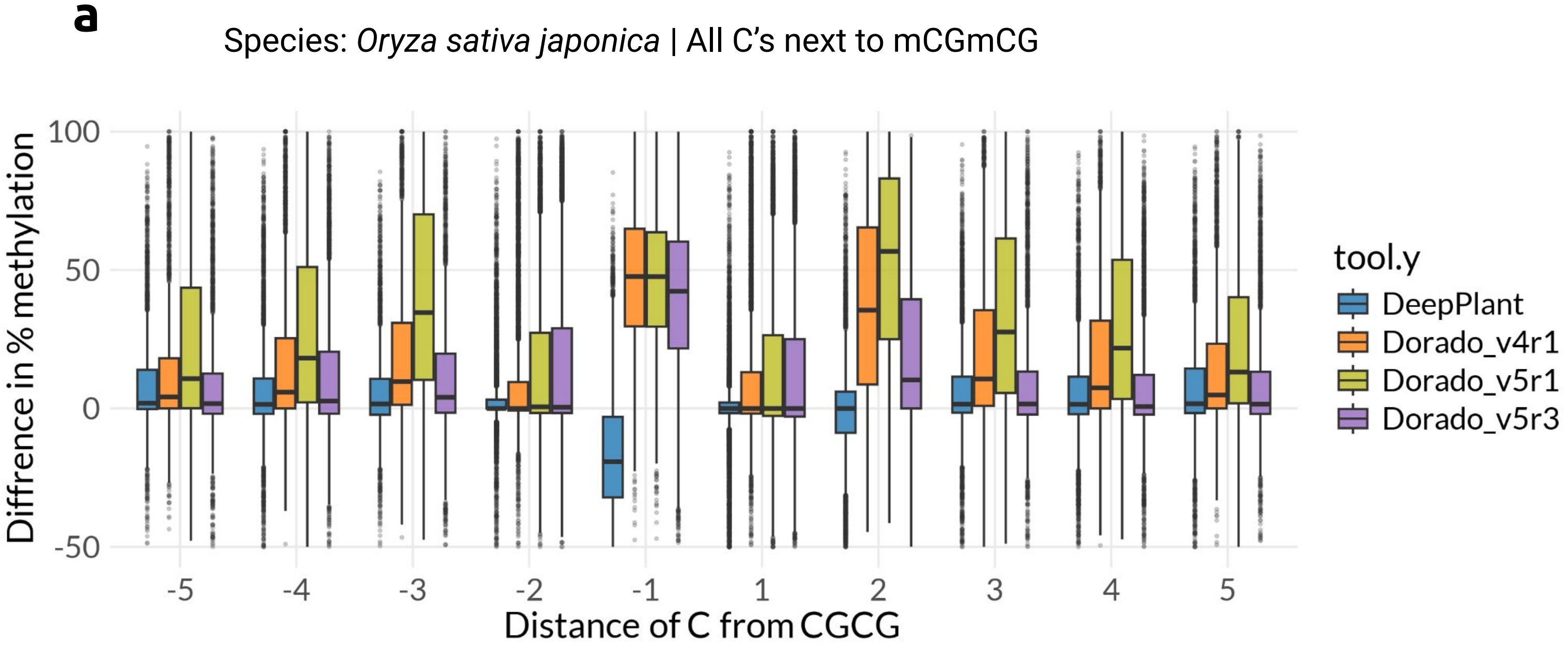

b

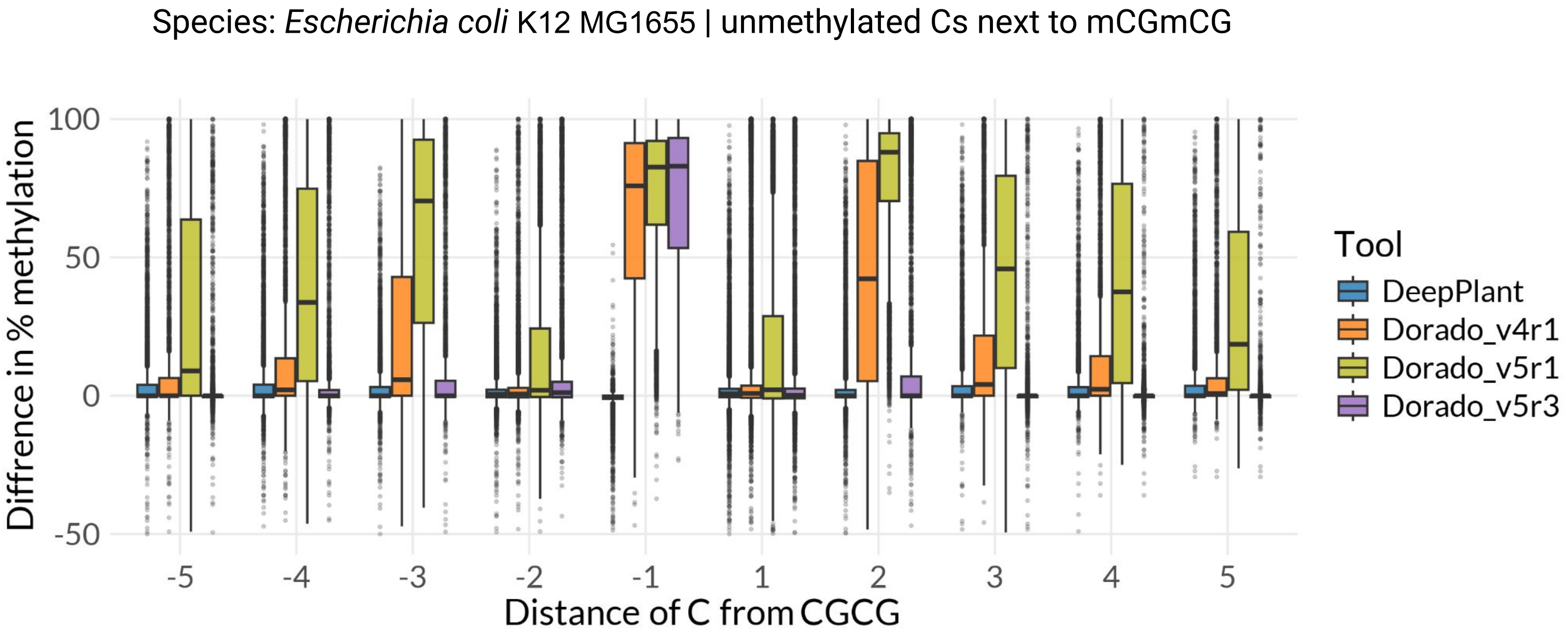

Supplementary Figure 19: Effect of two neighboring methylated CpGs. The difference in methylation calls for a cytosine as a function of its distance from CGCG sites where both CGs are methylated (mCGmCG). Negative values indicate underestimation of methylation near mCGmCG. EMSeq data is used as ground truth. Boxplot center lines indicate the median value, and lower and upper hinges represent the 25th and 75th percentiles, respectively. The whiskers denote 1.5x the interquartile range. a-b) Boxplots showing difference in methylation calls from the tool and EMSeq data for all Cs neighboring mCGmCG in *O.sativa* (a), unmethylated Cs neighboring mCGmCG in *E.coli* (b).

Supplementary Figure 20

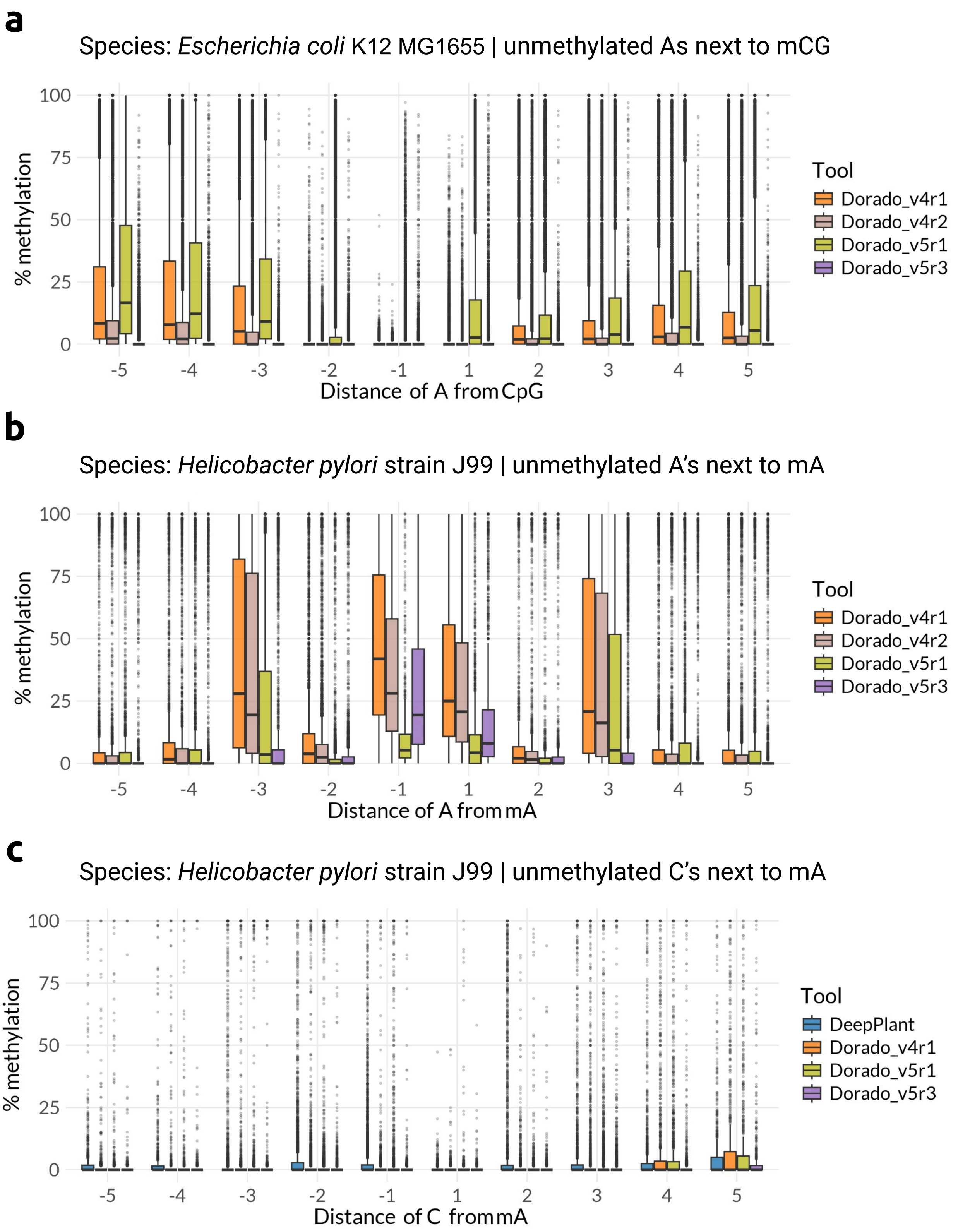

Supplementary Figure 20: Evaluating the effect of neighboring modifications on adenine methylation calling. Methylation percentage for a cytosine as a function of its distance from methylated sites. Boxplot center lines indicate the median value, and lower and upper hinges represent the 25th and 75th percentiles, respectively. The whiskers denote 1.5x the interquartile range. a-c) Boxplots showing methylation percentage from each tool for unmethylated As neighboring mCG sites in *E.coli* (a), unmethylated As neighboring mA sites in *E.coli* (b), unmethylated Cs neighboring mA sites in *H.pylori* J99 (c).

Supplementary Figure 21

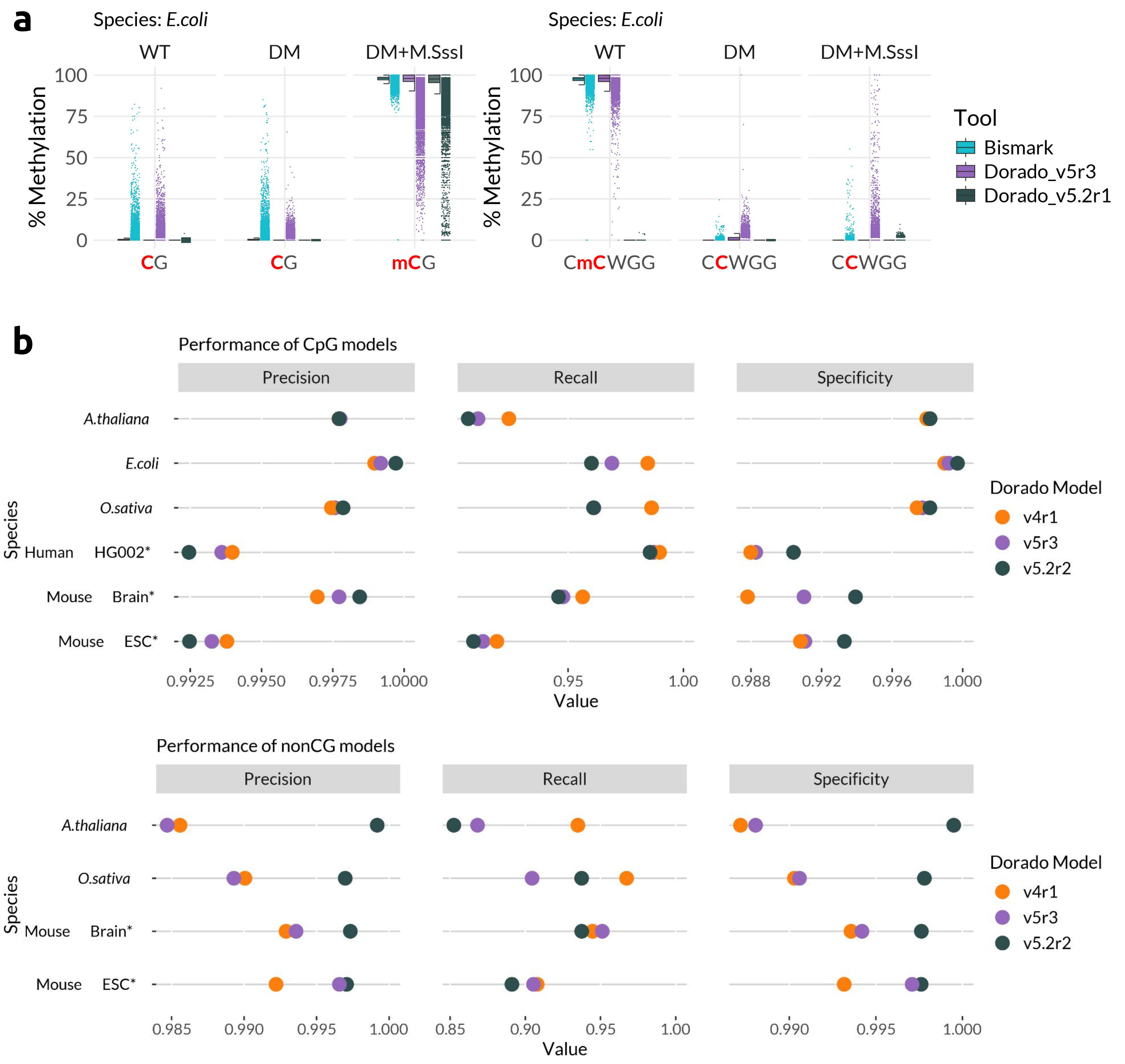

Supplementary Figure 21: Analysis of the new v5.2 models of Dorado in CpG and non-CG contexts. a) Performance of v5.2r1 models. Jitter-box plots depicting the percentage methylation in CG (n=693586) and CCWGG (n=24100) motifs in *E.coli* DM, WT and DM+M.SssI datasets. The profiled base is highlighted in red font. The methylation status of the bases is indicated with a preceding "m". The all context v5.2r1 model completely failed to report non CG methylation. In Jitter-box plots, boxplot center lines indicate the median value, and lower and upper hinges represent the 25th and 75th percentiles, respectively. The whiskers denote 1.5x the interquartile range. b) Read level evaluation of v5.2r2 CpG (up) and all-context C (down) models in comparison to v4r1 and v5r3 models. Precision (left), Recall (middle), Specificity (right) scores are depicted as colored dots on a number line (x-axis). Each line represents a dataset. The issue that was seen in v5.2r1 model has been rectified in the r2 models.

Supplementary Figure 22

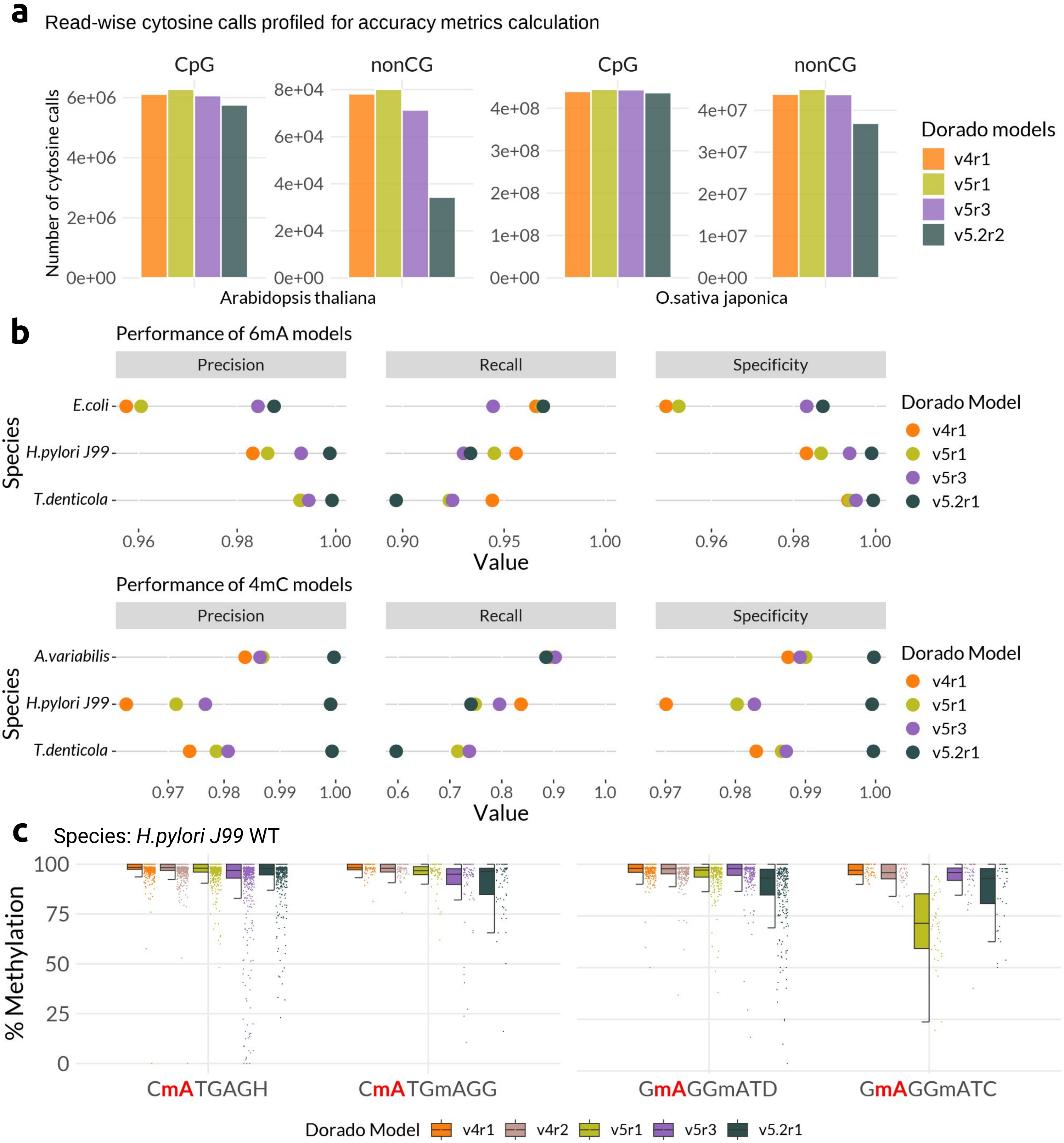

Supplementary Figure 22: Analysis of the new v5.2 models of Dorado. a) Bar plot showing number of read-wise cytosine calls profiled for accuracy metrics calculations across different dorado models for *A. thaliana* and *O. sativa* in both CpG and non-CG contexts, highlighting lower number of reads analyzed by the v5.2r2 model. b) Read level evaluation of v5.2r1 6mA (up) and 4mC (down) models in comparison to v4r1, v5r1 and v5r3 models. Precision (left), Recall (middle), Specificity (right) scores are depicted as colored dots on a number line (x-axis). Each line represents a dataset. c) Jitter-boxplots depicting the percent methylation of profiled As in the contexts CaTGAGN (CATGAGH, n=594; CATGAGG, n=76) and GaGGATN (GAGGATD, n=285; GAGGATC, n=44), in *H.pylori* str J99. In each context the mA preceding an umA is profiled, in contrast to profiling an mA preceding an mA. In Jitter-box plots, boxplot center lines indicate the median value, and lower and upper hinges represent the 25th and 75th percentiles, respectively. The whiskers denote 1.5x the interquartile range.

Supplementary Figure 23

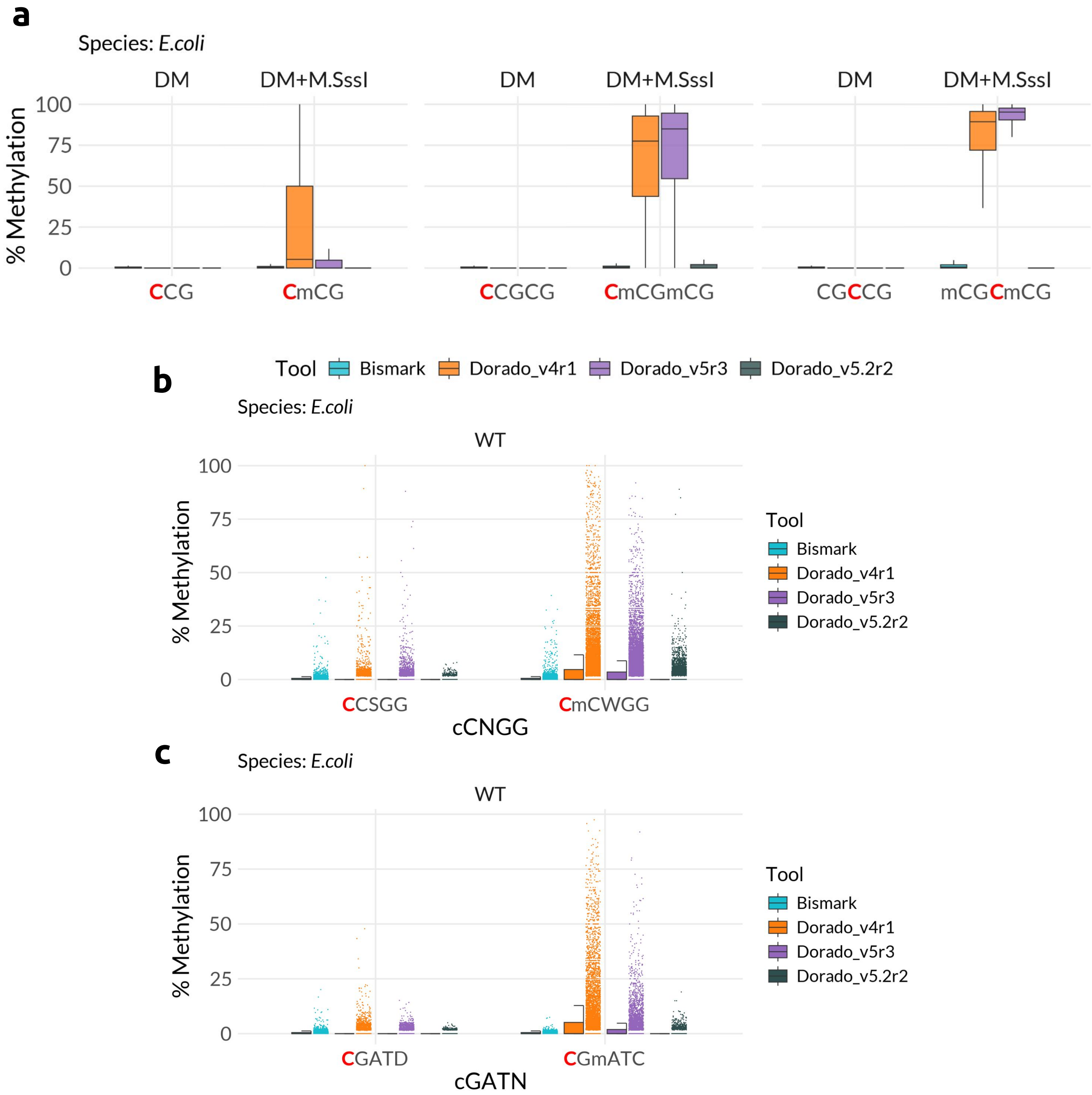

Supplementary Figure 23: Evaluating the effect of neighboring modifications on performance of v5.2 models, in comparison to v4r1 and v5r3 models in various contexts, using the *E.coli* dataset. Profiled base is shown in red. The methylation status of the bases is indicated with a preceding "m". a) Box plot depicting effect of mC on umC in various contexts in *E.coli* MSssI sample (CCG, n=173980; CCGCG, n=12594, CGCCG, n=20443). Boxplot center lines indicate the median value, and lower and upper hinges represent the 25th and 75th percentiles, respectively. The whiskers denote 1.5x the interquartile range. b) Jitter-box plot depicting effect of mC on umC in CCNGG context (CCSGG, n=18810, unmethylated; CCWGG, n=24100, methylated) in *E.coli* WT sample. In Jitter-box plots, boxplot center lines indicate the median value, and lower and upper hinges represent the 25th and 75th percentiles, respectively. The whiskers denote 1.5x the interquartile range. c) Jitter-box plot depicting effect of mA on umC in cGATN context (CGATD, n=37666, unmethylated; CGATC, n=10956, methylated) in *E.coli* WT sample. In Jitter-box plots, boxplot center lines indicate the median value, and lower and upper hinges represent the 25th and 75th percentiles, respectively. The whiskers denote 1.5x the interquartile range.

Supplementary Figure 24

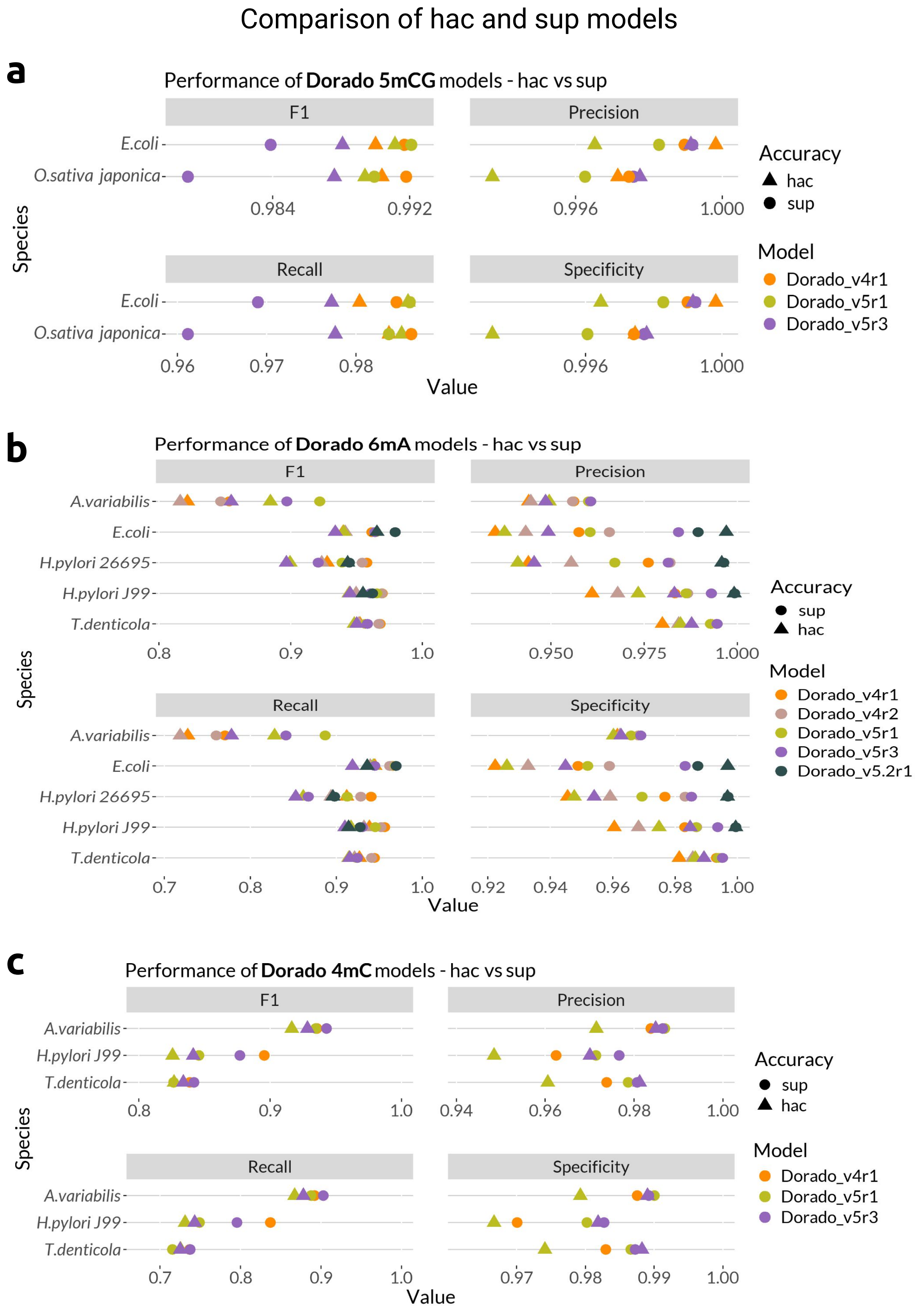

Supplementary Figure 24: Read-level comparison of Dorado 5mCG, 6mA and 4mC high-accuracy (hac) and super-accuracy (sup) models. The performance metrics, F1, Precision, Recall, and Specificity are plotted on a number line (x-axis) colored by tools. Each line represents a dataset. The sup models are plotted as triangles and the hac models as dots. a) The performance metrics for the Dorado 5mCG models of the rice and *E.coli* data are plotted on a number line. b) The performance metrics for both Dorado 6mA sup and hac models are compared across all bacterial species. c) The performance metrics for Dorado 4mC models for *A.variabilis*, *H.pylori* J99 and *T.denticola* data are plotted on a number line.

Supplementary Figure 25

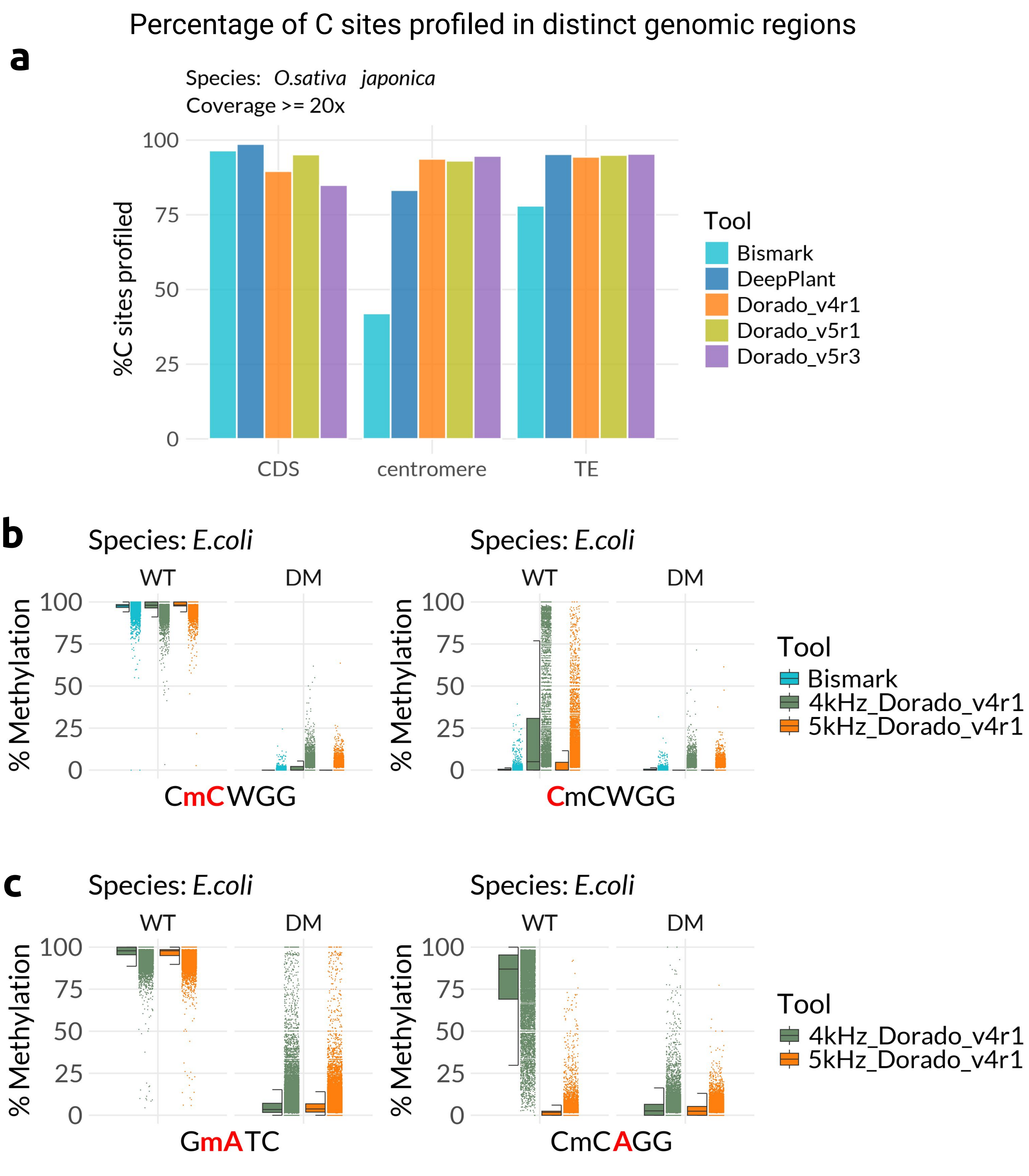

Supplementary Figure 25: Sites profiled by EMSeq and Nanopore, and susceptibility of 4kHz and 5Hz models to neighboring methylation. a) Percentage of cytosines in all contexts profiled (and covered by >=20 reads) in coding sequences, centromeres and transposable elements of the rice genome by different tools. The percentage of Cs profiled (y-axis) from the total genome at each distinct genomic region (x-axis) are shown as bar plots, colored by the methylation calling tools (Supplementary Data 12). b-c) Performance of Dorado 4kHz and 5kHz v4 models compared to the ground truth EMSeq data in the CCWGG (n=24100) (b) and GATC (n=38248) contexts (c) profiled in *E.coli* WT (methylated) and DM (unmethylated) samples (left). Effect of neighboring mC on unmethylated C (b), neighboring mC on unmethylated A (c) in CCWGG (CCAGG, n=12050) context in *E.coli* WT (methylated) and DM (unmethylated) samples (right). In Jitter-box plots, boxplot center lines indicate the median value, and lower and upper hinges represent the 25th and 75th percentiles, respectively. The whiskers denote 1.5x the interquartile range.

Supplementary Figure 26

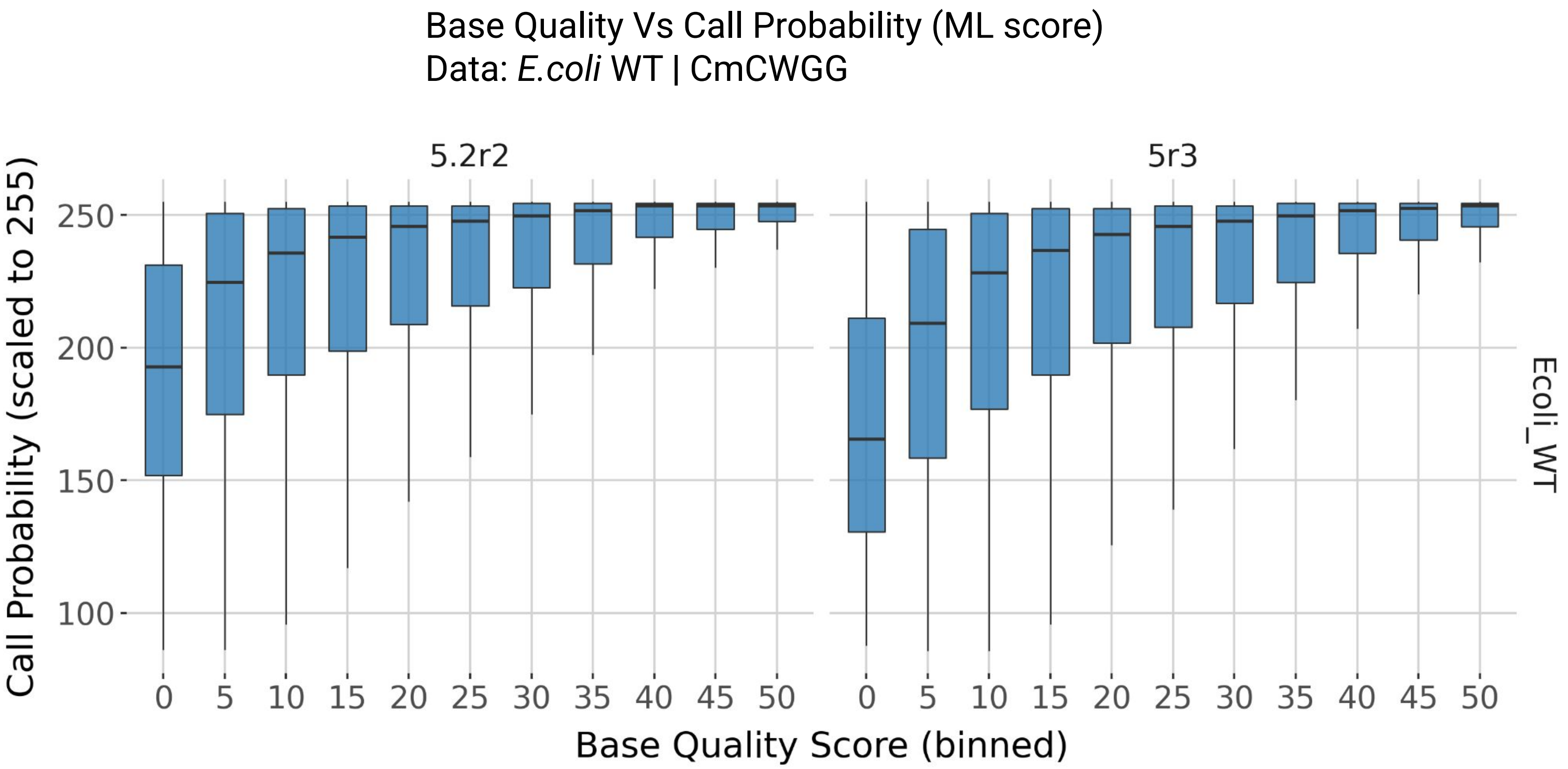

Supplementary Figure 26: Effect of base quality score (Phred score) on call probability (ML score) shown on *E.coli* WT dataset in CmCWGG context for Dorado v5.2r2 (left) and v5r3 (right) models. Call probability increases with increasing base quality. The modkit tool applies a threshold while selecting bases for methylation analysis which filters out low-confidence or calls arising from poor underlying Phred base quality scores. Boxplot center lines indicate the median value, and lower and upper hinges represent the 25th and 75th percentiles, respectively. The whiskers denote 1.5x the interquartile range.
