# Supplementary material for: Comprehensive benchmarking of tools for nanopore-based detection of DNA methylation
Source: Nat Commun. 2026 Jul 15;17:8685. doi: 10.1038/s41467-026-75183-6 (PMC13490465; doi:10.1038/s41467-026-75183-6)
Supplement: Supplementary file 2 [file 41467_2026_75183_MOESM2_ESM.pdf]

Description of Additional Supplementary Files

File Name: Supplementary Data 1
Description: Conversion efficiency of EMSeq datasets as measured on exogenous spike-ins.

File Name: Supplementary Data 2
Description: Nanopore and EMSeq datasets generated in this study and their quality metrics.

File Name: Supplementary Data 3
Description: Sequence contexts expected to carry methylation in various bacterial species used in this study.

File Name: Supplementary Data 4
Description: Representation of 9-mers and 11-mers covered by the datasets used for evaluation of 5mC.

File Name: Supplementary Data 5
Description: Overall abundance of various marks in the species and datasets used in the study.

File Name: Supplementary Data 6
Description: Versions of models used for calling methylation using various tools.

File Name: Supplementary Data 7
Description: Read level performance metrics and associated details across the tools and datasets.

File Name: Supplementary Data 8
Description: Effect of changing probability thresholds on various F1 metrics, and corresponding data loss.

File Name: Supplementary Data 9
Description: Site level evaluation metrics for 5mC calls across the datasets.

File Name: Supplementary Data 10
Description: Computational performance metrics of various tools utilized in this study.

File Name: Supplementary Data 11
Description: Reference genome versions used in the study.

File Name: Supplementary Data 12
Description: Cytosines profiled across genomic regions.
