# Supplementary material for: Comprehensive benchmarking of tools for nanopore-based detection of DNA methylation
Source: Nat Commun. 2026 Jul 15;17:8685. doi: 10.1038/s41467-026-75183-6 (PMC13490465; doi:10.1038/s41467-026-75183-6)
Supplement: Supplementary file 3 [file 41467_2026_75183_MOESM4_ESM.pdf]

nature portfolio

Corresponding author(s): Divya Tej Sowpati

Last updated by author(s): Jun 8, 2026

Reporting Summary

Nature Portfolio wishes to improve the reproducibility of the work that we publish. This form provides structure for consistency and transparency in reporting. For further information on Nature Portfolio policies, see our Editorial Policies and the Editorial Policy Checklist.

Statistics

For all statistical analyses, confirm that the following items are present in the figure legend, table legend, main text, or Methods section.

n/a	Confirmed	
☐	☒	The exact sample size (*n*) for each experimental group/condition, given as a discrete number and unit of measurement
☐	☒	A statement on whether measurements were taken from distinct samples or whether the same sample was measured repeatedly
☒	☐	The statistical test(s) used AND whether they are one- or two-sided<br>*Only common tests should be described solely by name; describe more complex techniques in the Methods section.*
☒	☐	A description of all covariates tested
☒	☐	A description of any assumptions or corrections, such as tests of normality and adjustment for multiple comparisons
☐	☒	A full description of the statistical parameters including central tendency (e.g. means) or other basic estimates (e.g. regression coefficient) AND variation (e.g. standard deviation) or associated estimates of uncertainty (e.g. confidence intervals)
☒	☐	For null hypothesis testing, the test statistic (e.g. *F*, *t*, *r*) with confidence intervals, effect sizes, degrees of freedom and *P* value noted<br>*Give P values as exact values whenever suitable.*
☒	☐	For Bayesian analysis, information on the choice of priors and Markov chain Monte Carlo settings
☒	☐	For hierarchical and complex designs, identification of the appropriate level for tests and full reporting of outcomes
☐	☒	Estimates of effect sizes (e.g. Cohen's *d*, Pearson's *r*), indicating how they were calculated

Our web collection on statistics for biologists contains articles on many of the points above.

Software and code

Policy information about availability of computer code

Data collection

The pod5 files for ONT data were basecalled using Dorado v0.5.3 for v4 models (4kHz and 5kHz), Dorado v0.9.1 for v5 models, and v1.0.0 for v5.2 models available on ONT's Dorado GitHub repository (https://github.com/nanoporetech/dorado). Raw data of Enzymatic Methyl Seq (EMSeq) was converted from BCL to FASTQ format using bcl2fastq (Illumina).

Data analysis

Scripts used for processing and plotting the data are available at https://github.com/SowpatiLab/ONT_Methylation_Benchmarking

EMSeq raw read quality was checked using FastQC (v0.12.1) and Cutadapt (v4.8). Reference alignment and methylation extraction were performed using Bismark (v0.24.2). The modbam files that were generated by Dorado were processed using Modkit v0.4.4 (v0.5.1 for Dorado v5.2 models) (https://github.com/nanoporetech/modkit) for testing ONT models.

For comparison of Dorado models, the following combinations of models were used:

4kHz v4r1 Dorado:
Basecalling model: res_dna_r10.4.1_e8.2_400bps_sup@v4.0.1
5mC methylation model: res_dna_r10.4.1_e8.2_400bps_sup@v4.0.1_5mC@v2

5kHz v4r1 Dorado
Basecalling model:dna_r10.4.1_e8.2_400bps_sup@v4.3.0
5mC methylation model: dna_r10.4.1_e8.2_400bps_sup@v4.3.0_5mC_5hmC@v1
5mCG methylation model: dna_r10.4.1_e8.2_400bps_sup@v4.3.0_5mCG_5hmCG@v1

4mC methylation model: res_dna_r10.4.1_e8.2_400bps_sup@v4.3.0_4mC_5mC@v1
6mA methylation model: dna_r10.4.1_e8.2_400bps_sup@v4.3.0_6mA@v2

5kHz v5r1 Dorado
Basecalling model:dna_r10.4.1_e8.2_400bps_sup@v5.0.0
5mC methylation model: dna_r10.4.1_e8.2_400bps_sup@v5.0.0_5mC_5hmC@v1
5mCG methylation model: dna_r10.4.1_e8.2_400bps_sup@v5.0.0_5mCG_5hmCG@v1
4mC methylation model: dna_r10.4.1_e8.2_400bps_sup@v5.0.0_4mC_5mC@v1
6mA methylation model: dna_r10.4.1_e8.2_400bps_sup@v5.0.0_6mA@v1

5kHz v5r3 Dorado
Basecalling model:dna_r10.4.1_e8.2_400bps_sup@v5.0.0
5mC methylation model: dna_r10.4.1_e8.2_400bps_sup@v5.0.0_5mC_5hmC@v3
5mCG methylation model: dna_r10.4.1_e8.2_400bps_sup@v5.0.0_5mCG_5hmCG@v3
4mC methylation model: dna_r10.4.1_e8.2_400bps_sup@v5.0.0_4mC_5mC@v3
6mA methylation model: dna_r10.4.1_e8.2_400bps_sup@v5.0.0_6mA@v3

5kHz v5.2r1 Dorado
Basecalling model:dna_r10.4.1_e8.2_400bps_sup@v5.2.0
5mC methylation model: dna_r10.4.1_e8.2_400bps_sup@v5.2.0_5mC_5hmC@v1
5mCG methylation model: dna_r10.4.1_e8.2_400bps_sup@v5.2.0_5mCG_5hmCG@v1
4mC methylation model: dna_r10.4.1_e8.2_400bps_sup@v5.2.0_4mC_5mC@v1
6mA methylation model: dna_r10.4.1_e8.2_400bps_sup@v5.2.0_6mA@v1

5kHz v5.2r2 Dorado
Basecalling model:dna_r10.4.1_e8.2_400bps_sup@v5.2.0
5mC methylation model: dna_r10.4.1_e8.2_400bps_sup@v5.2.0_5mC_5hmC@v2
5mCG methylation model: dna_r10.4.1_e8.2_400bps_sup@v5.2.0_5mCG_5hmCG@v2

For comparison with other tools, Dorado was run with the basecalling model alone, with the '--emit-moves' flag and '--min-qscore' set to 10; the resultant BAMs were further processed by each of the other tools for methylation identification. The following tool and model combinations were used to evaluate the other tools.

DeepMod2 (https://github.com/WGLab/DeepMod2)

5kHz data:
dorado basecalling model:dna_r10.4.1_e8.2_400bps_sup@v5.0.0
deepmod2 biLSTM model: bilstm_r10.4.1_5khz_v5.0
deepmod2 transformer model: transformer_r10.4.1_4khz_v5.0

F5c v1.5 (https://github.com/hasindu2008/f5c)

Rockfish r10.9.1 (https://github.com/lbcb-sci/rockfish)
rf_5kHz.ckpt
DeepPlant (https://github.com/xiaochuanle/DeepPlant)
both_bilstm.b13_s15_epoch8.chh
both_bilstm.b51_s15_epoch9.chg
both_bilstm.b51_s15_epoch8.cpg
DeepBAM (https://github.com/xiaochuanle/DeepBAM)
LSTM_20240524_newfeature_script_b9_s15_epoch25_accuracy0.9742.pt

After each tool is run, a readwise methylation file is generated, each of which is then aggregated by scripts provided by their respective tool, producing a per-site methylation file.

For manuscripts utilizing custom algorithms or software that are central to the research but not yet described in published literature, software must be made available to editors and reviewers. We strongly encourage code deposition in a community repository (e.g. GitHub). See the Nature Portfolio guidelines for submitting code & software for further information.

Data

Policy information about availability of data

All manuscripts must include a data availability statement. This statement should provide the following information, where applicable:
- Accession codes, unique identifiers, or web links for publicly available datasets
- A description of any restrictions on data availability
- For clinical datasets or third party data, please ensure that the statement adheres to our policy

Raw signal data in POD5 format, as well as processed data is available via the Registry of Open Data hosted on AWS at https://registry.opendata.aws/ont_basemod_data/. Reference aligned Modbam files generated in this study are deposited to the NCBI SRA under the BioProject number PRJNA1172918. Nextflow and Snakemake pipelines to reproduce the analysis or benchmark new data, and code used to process and visualize data from this work is available at https://github.com/SowpatiLab/ONT_Methylation_Benchmarking.

Other data sources:
O.sativa GTF file: https://ftp.ncbi.nlm.nih.gov/genomes/all/GCF/034/140/825/GCF_034140825.1_ASM3414082v1/GCF_034140825.1_ASM3414082v1_genomic.gtf.gz
GRCm39/mm39 assembly gene annotations: https://genome.ucsc.edu/cgi-bin/hgTables
Rerio repository: https://github.com/nanoporetech/rerio
HG002 WGBS data: s3://ont-open-data/gm24385_mod_2021.09/bisulphite/bismark/
Bacterial PacBio data: https://downloads.pacbcloud.com/public/dataset/2021-11-Microbial-96plex/demultiplexed-reads/
Source data are provided with this paper.

Research involving human participants, their data, or biological material

Policy information about studies with human participants or human data. See also policy information about sex, gender (identity/presentation), and sexual orientation and race, ethnicity and racism.

Reporting on sex and gender	NA
Reporting on race, ethnicity, or other socially relevant groupings	NA
Population characteristics	NA
Recruitment	NA
Ethics oversight	NA

Note that full information on the approval of the study protocol must also be provided in the manuscript.

Field-specific reporting

Please select the one below that is the best fit for your research. If you are not sure, read the appropriate sections before making your selection.

[x] Life sciences [ ] Behavioural & social sciences [ ] Ecological, evolutionary & environmental sciences

For a reference copy of the document with all sections, see nature.com/documents/nr-reporting-summary-flat.pdf

Life sciences study design

All studies must disclose on these points even when the disclosure is negative.

Sample size	As the purpose of this study was to benchmark various tools, we have used multiple species under various conditions (mutants lacking methylation, whole genome amplification, in vitro methylated). Hence, a sample size is not directly applicable.
Data exclusions	Reads under a Phred quality of 10, reads of length lesser than 500BP and non primary aligned reads were removed. Genomic sites in the modbed file with coverage <20 were filtered out for all analyses, except when the effect of coverage was analysed.
Replication	Raw signal data is available via the Registry of Open Data hosted on AWS at https://registry.opendata.aws/ont_basemod_data/. Nextflow and snakemake pipelines to reproduce our analysis are available at https://github.com/SowpatiLab/ONT_Methylation_Benchmarking. Processed BAM files are available on RODA and also are submitted to NCBI SRA (PRJNA1172918), and all code used to perform analysis and generate figures is available on the git repository: https://github.com/SowpatiLab/ONT_Methylation_Benchmarking.
Randomization	As the study relied on various species and conditions to benchmark the tools, randomization was not required
Blinding	All computational methods were blinded to the ground-truth. The output of the methods was compared directly to the available ground-truth

Reporting for specific materials, systems and methods

We require information from authors about some types of materials, experimental systems and methods used in many studies. Here, indicate whether each material, system or method listed is relevant to your study. If you are not sure if a list item applies to your research, read the appropriate section before selecting a response.

Materials & experimental systems

n/a	Involved in the study
☒	☐ Antibodies
☐	☒ Eukaryotic cell lines
☒	☐ Palaeontology and archaeology
☒	☐ Animals and other organisms
☒	☐ Clinical data
☒	☐ Dual use research of concern
☒	☐ Plants

Methods

n/a	Involved in the study
☒	☐ ChIP-seq
☒	☐ Flow cytometry
☒	☐ MRI-based neuroimaging

Eukaryotic cell lines

Policy information about cell lines and Sex and Gender in Research

Cell line source(s)	GM24385 (HG002) from the Genome-in-a-Bottle consortium was sourced from Coriell Institute for Medical Research.
Authentication	The cell line was not specifically authenticated as it was procured from Coriell Institute for Medical Research.
Mycoplasma contamination	Cell line was not tested for Mycoplasma contamination.
Commonly misidentified lines (See ICLAC register)	No commonly misidentified cell lines were used.

Plants

Seed stocks	*Report on the source of all seed stocks or other plant material used. If applicable, state the seed stock centre and catalogue number. If plant specimens were collected from the field, describe the collection location, date and sampling procedures.*
Novel plant genotypes	*Describe the methods by which all novel plant genotypes were produced. This includes those generated by transgenic approaches, gene editing, chemical/radiation-based mutagenesis and hybridization. For transgenic lines, describe the transformation method, the number of independent lines analyzed and the generation upon which experiments were performed. For gene-edited lines, describe the editor used, the endogenous sequence targeted for editing, the targeting guide RNA sequence (if applicable) and how the editor was applied.*
Authentication	*Describe any authentication procedures for each seed stock used or novel genotype generated. Describe any experiments used to assess the effect of a mutation and, where applicable, how potential secondary effects (e.g. second site T-DNA insertions, mosiacism, off-target gene editing) were examined.*
