# Supplementary material for: Comprehensive benchmarking of tools for nanopore-based detection of DNA methylation
Source: Nat Commun. 2026 Jul 15;17:8685. doi: 10.1038/s41467-026-75183-6 (PMC13490465; doi:10.1038/s41467-026-75183-6)
Supplement: Supplementary file 4 [file 41467_2026_75183_MOESM5_ESM.pdf]

nature portfolio

Peer Review File

Comprehensive benchmarking of tools for nanopore-based detection of DNA methylation

Corresponding Author: Dr Divya Tej Sowpati

This manuscript has been previously reviewed at another journal. This document only contains information relating to versions considered at Nature Communications.

This file contains all reviewer reports in order by version, followed by all author rebuttals in order by version.

Version 1:

Reviewer comments:

Reviewer #1

(Remarks to the Author)
0) The authors argue their snakemake can be used easily, but a cursory examination of their smk file still reveals hardcoded absolute paths that seem likely to be computer specific. This is not something that is dockerized or truly portable as is. An improvement of this to a more robust tooling would be really valuable.

3) They created their own, custom, KO strain. This strain is not easily obtained or reproducible. If a WGA is an easy replacement, why not just use WGA? The raw data being available is a plus, but if the sequencing chemistry changes, a rerun would be needed.

4a) The authors should at least report this point, and I don't think its' completely clear the effects of sequence, even if marginal, on error rate. At the very least we know that bases in the motor protein ~10 b upstream can have an impact. And we know the NN is using a large window, even if we don't expect that that should make any biophysical difference to the current. The authors should at least illuminate this point to non-aware readers.

9b) The intent of the comment is to reinforce that mammalian DNA (at CGs) is unlikely to be 100% methylated. And that if the authors are trying to demonstrate the strength of different basecallers and platforms. The authors are looking at "fully methylated" locations in mouse brain, human HG002, mouse ESC in Figure 2A.

10B) You point it out for deepbam and deep plant, but it seems pretty ubiquitous? And you didn't look at or identify if there were specific contexts where this happened, or if it was just happening generally? If the authors are trying to make a tool that's useful for the community to compare pluses and minuses of basecalling/chemistry metrics, that's perhaps the most useful approach?

10C) The authors are arguing this is due to false positive calls by nanopore models and not random dispersion, but . . . how are they proving that? With the exception of the plant samples (and maybe the mouse brain) the levels of non-CG are really low. I get that the RMSE and MAE seem more robust to differences in abundance, but I think really only the plant sample is a useful one to use for non-CG exploration of the ones they looked at here.

10D) Sure I'm not arguing that analyzing nanopore data isn't valuable, I'm saying that focusing the analysis on the available models just with stats, without insight into the difficult regions, is not valuable. The data on the AWS open data registry and anekmake is the right answer. But unfortunately I found the code they provided in the github had too many hardcoded paths in a cursory examination. If the goal of this work is to provide a tool for benchmarking that's easily runnable, it's not yet achieved as stated.

20B) I agree that they showed read filtering is useful - but I do not see in the manuscript where they instead attempted to apply a per base quality filter instead? Was this done? If so, it should be featured more clearly.

(Remarks on code availability)
Code limited utility, not easy to just run directly on other systems.

Reviewer #3

(Remarks to the Author)
The revised manuscript addresses my remaining comments.

There is one small addition to the new version that should be clarified:
Line 432/433: 'However, they profiled fewer reads compared to the older versions (Table S7)' I was not able to get this information from the supplementary table and don't understand what point is being made. This should be clarified and perhaps better presented in a different way, eg a bar graph of total read counts profiled in the supplementary figure.

(Remarks on code availability)

Version 2:

Reviewer comments:

Reviewer #1

(Remarks to the Author)
0 & 10D) Read the workflow output this round. Final files in output/meta/{tool}/ are methylBEDs with metadata columns — useful, but not a benchmark. The scoring lives in performance_metrics_full_data.sh, which states: "This is a scaffold script... not intended to be used as is. The variables need to be adapted." Below that are awk one-liners with hardcoded column indices, motif by motif. Figures come from 12 bespoke R scripts.

So there's no reusable scoring layer. To benchmark Dorado v6 next quarter, someone has to rewrite that scaffold. That's why the contribution is limited — not because the data isn't valuable, but because the benchmark part doesn't exist as a tool.

What I strongly suggest the authors should do - have the output from the nextflow not just be the methylBED but also F1/precision/recall/RMSE per tool/motif/context. Your awk is 90% of it. Refactor it into something reusable and this becomes a much stronger community resource.

3) Agree to disagree. The authors are clear on what they are doing.

4a) I acquiesce on this point, I still feel like given the topic this could//should have been expanded about the window of signal the basecaller is using.

9b) Still not addressed. State the threshold and coverage you used to call locations "fully methylated" in HG002, mouse brain, and mouse ESC. Composite datasets don't answer it.

10B) Ok

10C) The FP/FN bar chart is good. Please add this to the supplement.

20B) The Qscore plot answers the question - make sure this is clear and please add this to the supplement.

(Remarks on code availability)

Open Access This Peer Review File is licensed under a Creative Commons Attribution 4.0 International License, which permits use, sharing, adaptation, distribution and reproduction in any medium or format, as long as you give appropriate credit to the original author(s) and the source, provide a link to the Creative Commons license, and indicate if changes were made.
In cases where reviewers are anonymous, credit should be given to 'Anonymous Referee' and the source.
The images or other third party material in this Peer Review File are included in the article's Creative Commons license, unless indicated otherwise in a credit line to the material. If material is not included in the article's Creative Commons license and your intended use is not permitted by statutory regulation or exceeds the permitted use, you will need to obtain permission directly from the copyright holder.
To view a copy of this license, visit https://creativecommons.org/licenses/by/4.0/

Response to Reviewer Comments

We thank the reviewers for dedicating their time and providing further comments that helped us refine our work. We have addressed all their comments. In particular:

- Improved the reproducibility of our pipelines by dockerizing various tools and integrating them into the workflow.
- Added a detailed tutorial to reproduce the workflow on AWS.
- Made changes to the manuscript to incorporate other suggestions to improve the clarity of our findings.

A point by point response to the comments is provided below in blue font.

Reviewer #1 (Remarks to the Author):

0) The authors argue their snakemake can be used easily, but a cursory examination of their smk file still reveals hardcoded absolute paths that seem likely to be computer specific. This is not something that is dockerized or truly portable as is. An improvement of this to a more robust tooling would be really valuable.

Response: Thank you for pointing out that there was a hardcoded path in our snakemake file. This was an oversight from us and we have since corrected this error.

We would like to further clarify that since the last revision, we have focussed more on Nextflow pipelines than the snakemake files due to the fact that Nextflow makes it easier to download and deploy pipelines via containers.

Having said this, we have now extensively tested our pipelines on multiple systems and settings. In addition, we have provided detailed README on our GitHub repository to aid users in running the workflows. We also created the docker containers of various tools compatible with these workflows and uploaded them on dockerhub (https://hub.docker.com/r/sowpati/ont-methylation-benchmarking). Finally, we have also created a tutorial specifically to run our workflow on AWS (https://github.com/SowpatiLab/ont-basemod-benchmark-data/blob/main/tutorial_workflow.md).

We believe these changes address the reproducibility and code/data availability concerns raised.

3) They created their own, custom, KO strain. This strain is not easily obtained or reproducible. If a WGA is an easy replacement, why not just use WGA? The raw data being available is a plus, but if the sequencing chemistry changes, a rerun would be needed.

Response: As clarified in one of our earlier response letters, the main reason we used this KO strain in lieu of WGA was so that the strain lacks canonical *dcm* and *dam* methylation, but other

sites are unaffected. This lets us rule out any bias that could be happening due to WGA (we did use WGA for *H.pylori* for completeness), and more importantly, this sample gave us the earliest insight into the effect of neighboring methylation - which would have been impossible to study using a WGA sample.

Also, like explained before, this contributes to one out of many diverse datasets used in the study, and including/excluding/replacing with WGA does not change the overall conclusions we draw from our work. Furthermore, to facilitate reproducibility for other researchers, we have deposited the signal level data of the WGA sample as well, on AWS RODA.

4a) The authors should at least report this point, and I don't think its' completely clear the effects of sequence, even if marginal, on error rate. At the very least we know that bases in the motor protein ~10 b upstream can have an impact. And we know the NN is using a large window, even if we don't expect that that should make any biophysical difference to the current. The authors should at least illuminate this point to non-aware readers.

Response: We agree with the reviewer that pointing this out would be helpful for the reader. In the revision, we have now incorporated this change, where we talk about the effect of neighboring methylation (lines 375-379).

9b) The intent of the comment is to reinforce that mammalian DNA (at CGs) is unlikely to be 100% methylated. And that if the authors are trying to demonstrate the strength of different basecallers and platforms. The authors are looking at "fully methylated" locations in mouse brain, human HG002, mouse ESC in Figure 2A.

Response: Thank you for providing the clarification. Like explained in our previous response letter, our datasets span both motif-driven bacterial systems, as well as eukaryotic data where the methylation levels are a continuum with many locations fully methylated. Our conclusions are drawn based on the composite of these diverse species, and our figures depict the results on all relevant datasets.

10B) You point it out for deepbam and deep plant, but it seems pretty ubiquitous? And you didn't look at or identify if there were specific contexts where this happened, or if it was just happening generally? If the authors are trying to make a tool that's useful for the community to compare pluses and minuses of basecalling/chemistry metrics, that's perhaps the most useful approach?

Response: We pointed this out for deepbam and deepplant in the response letter. Our kmer/context analysis was done for all tools in both CpG and non-CG contexts (Fig 3d, S9, lines 309-313, 332-337). In addition to the kmers and specific contexts, we also talked about other reasons due to which models were not accurate - such as low levels of target modification, and presence of confounding modifications nearby.

10C) The authors are arguing this is due to false positive calls by nanopore models and not random dispersion, but . . . how are they proving that? With the exception of the plant samples

(and maybe the mouse brain) the levels of non-CG are really low. I get that the RMSE and MAE seem more robust to differences in abundance, but I think really only the plant sample is a useful one to use for non-CG exploration of the ones they looked at here.

Response: The propensity for false positives was discussed in great detail in our response letter for R1 (as a response to comment 15 by Reviewer 1 and comment about original Figure 2H by Reviewer 3). The bias towards FPs is evident from the site-level heatmaps we plotted for various Dorado models. To reiterate this point, we have plotted below the number of locations reported to be <5% methylated by EMSeq and >25% by nanopore models (left most columns of the heatmap representing FPs) and vice versa - <5% by nanopore models and >25% by EMSeq (bottom most rows of the heatmap, representing FNs), using the HG002 dataset. As can be seen below, the number of FN is a tiny fraction compared to FP.

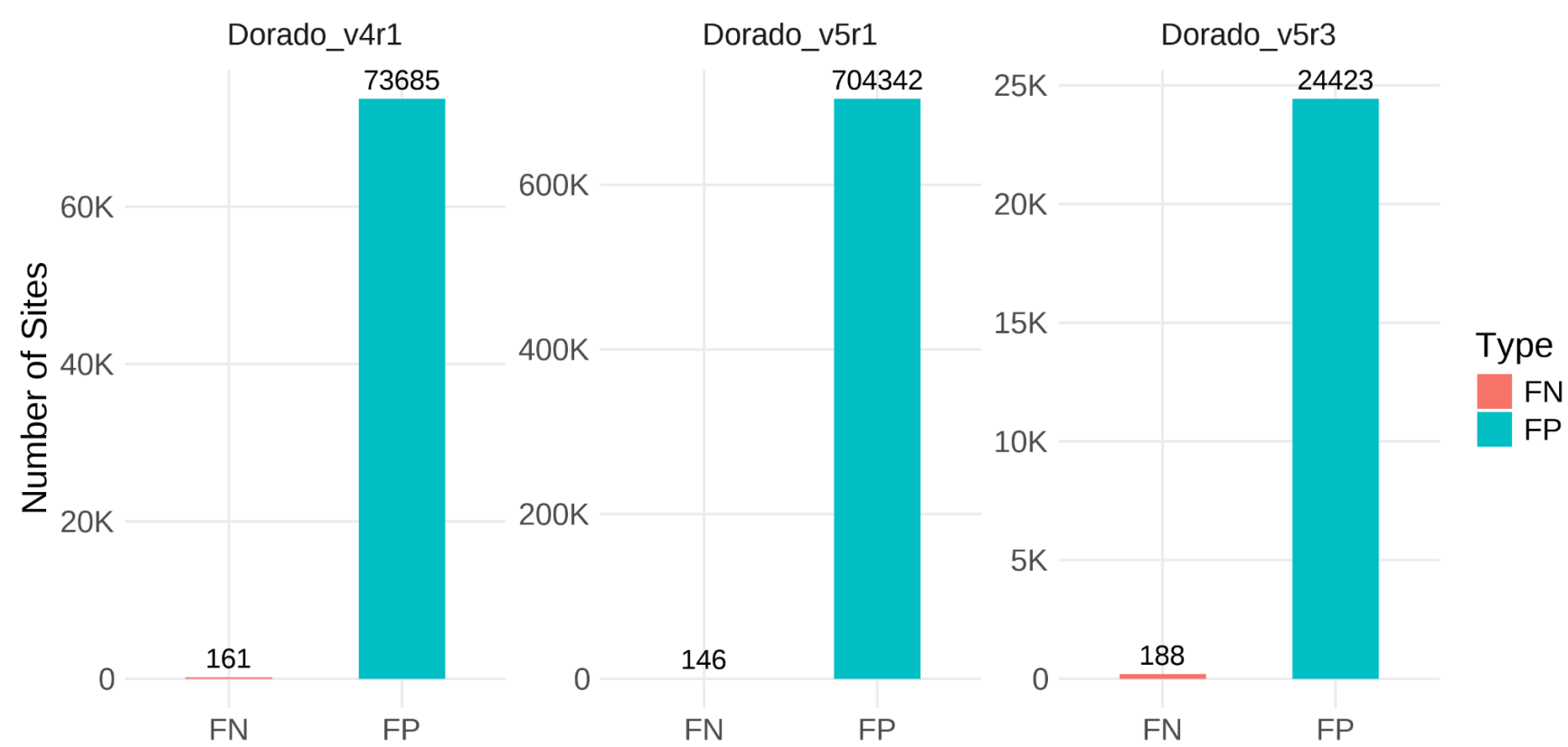

We also agree that the Rice sample has the best non-CG representation, which is why most of our non-CG methylation analysis is done on that data. But, we did not stop there because not all researchers are going to study samples with abundant non-CG methylation. Our goal was to also provide guidelines to people aiming to study this in less abundant data, and what pitfalls may arise due to that. In this pursuit, we show that the number of FPs is inversely proportional to the overall abundance of non-CG methylation (Fig 3e, Table S5). More importantly, we provide a practical workaround: using a stringent modbase threshold reduces FPs while not increasing too many FNs, as demonstrated using the Mouse ESC dataset (Fig S12).

We believe this analysis is helpful to a broad range of researchers using nanopore sequencing studying DNA methylation, as they may not always be restricting themselves to studying just the abundant modifications.

10D) Sure I'm not arguing that analyzing nanopore data isn't valuable, I'm saying that focusing the analysis on the available models just with stats, without insight into the difficult regions, is not valuable. The data on the AWS open data registry and anekmake is the right answer. But unfortunately I found the code they provided in the github had too many hardcoded paths in a cursory examination. If the goal of this work is to provide a tool for benchmarking that's easily runnable, it's not yet achieved as stated.

Response: Thank you for acknowledging our efforts in benchmarking and making the data/pipelines more widely accessible to other researchers. As also explained in the response to another comment above, we have now corrected the hard path error in our .smk file. We have also extensively tested these workflows on various computational environments to ensure they run as expected.

20B) I agree that they showed read filtering is useful - but I do not see in the manuscript where they instead attempted to apply a per base quality filter instead? Was this done? If so, it should be featured more clearly.

Response: Thank you for this comment. To clarify, we did not do a base level filter - this happens by default due to ML score thresholds, as explained further in this response. Our response in the earlier letter was regarding the comment by the reviewer that the ML score given by the model already incorporates the base level Phred score information. We acknowledge(d) that the reviewer is correct about that statement, as we further confirm with the plot below.

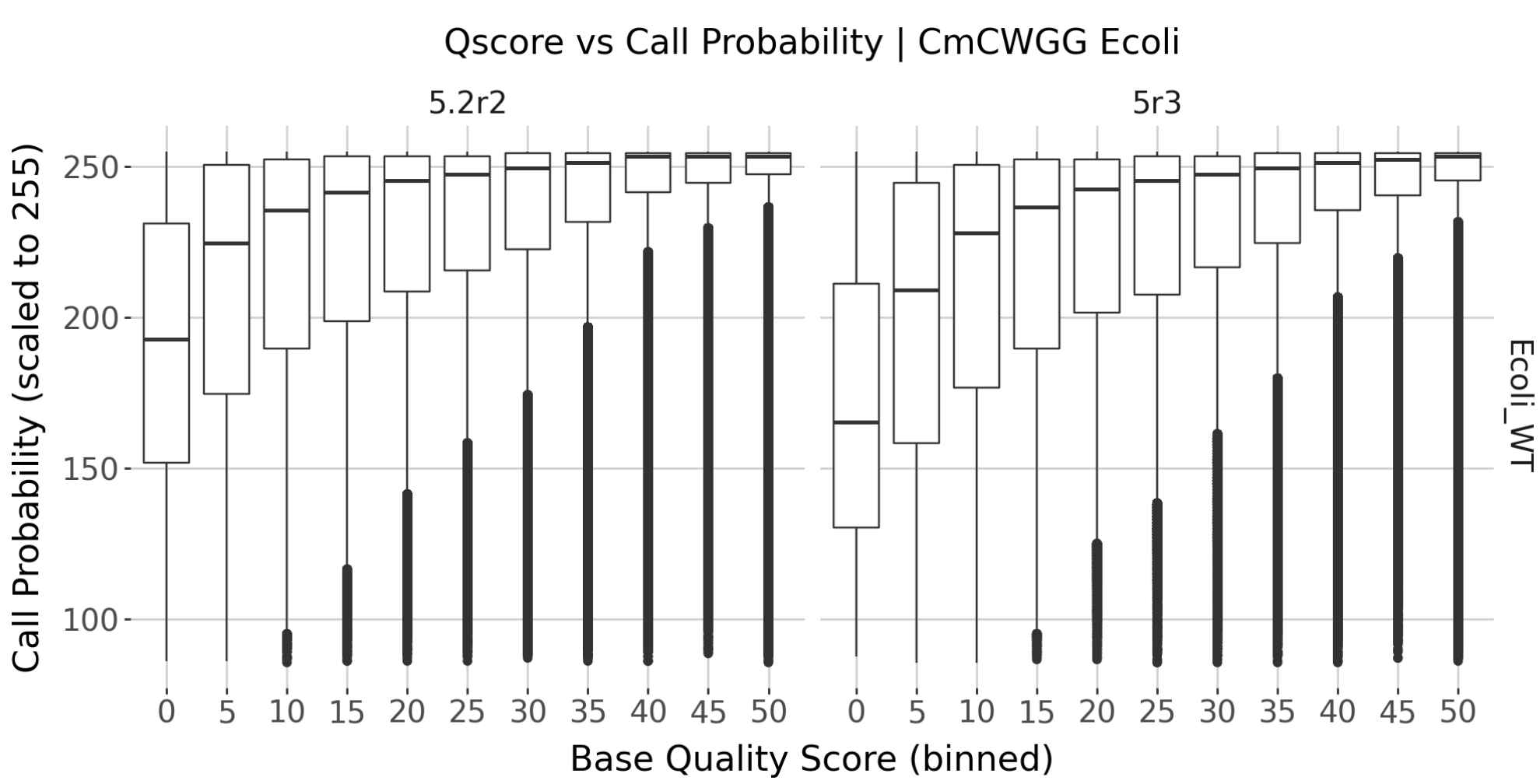

This would mean that, once modkit uses a threshold when considering the bases to calculate methylation, “bad calls” which are bad due to underlying poor Phred score at a base level would already be filtered out. Ideally, this should be enough to negate the effect of poor Phred quality. But, we show that despite this default filtering at base level, filtering for reads which are of overall lower quality improves the accuracy of various models, and hence suggest applying this filter to users in their analysis.

Reviewer #1 (Remarks on code availability):

Code limited utility, not easy to just run directly on other systems.

Response: As detailed in responses to other relevant comments above, we have now improved our NextFlow/snakemake pipelines and the corresponding documentation to make our work easily reproducible by others. We have also provided a tutorial to deploy the pipelines directly on AWS, leveraging our RODA datasets.

Reviewer #3 (Remarks to the Author):

The revised manuscript addresses my remaining comments.

There is one small addition to the new version that should be clarified:
Line 432/433: 'However, they profiled fewer reads compared to the older versions (Table S7)' I was not able to get this information from the supplementary table and don't understand what point is being made. This should be clarified and perhaps better presented in a different way, eg a bar graph of total read counts profiled in the supplementary figure.

Response: Thank you for the positive assessment of our revision. We apologize for not explaining the “profiled fewer reads” part clearly. We agree with the reviewer and thank them for the suggestion that depicting this as a bar chart would be a far better presentation than expecting a reader to fetch this information out from the table. Hence, we added a bar chart of reads profiled by various models as Supplementary Figure S22a, which is also pasted below for your reference. We have also reworked the corresponding section in the manuscript slightly to make it flow better (lines 436-438).

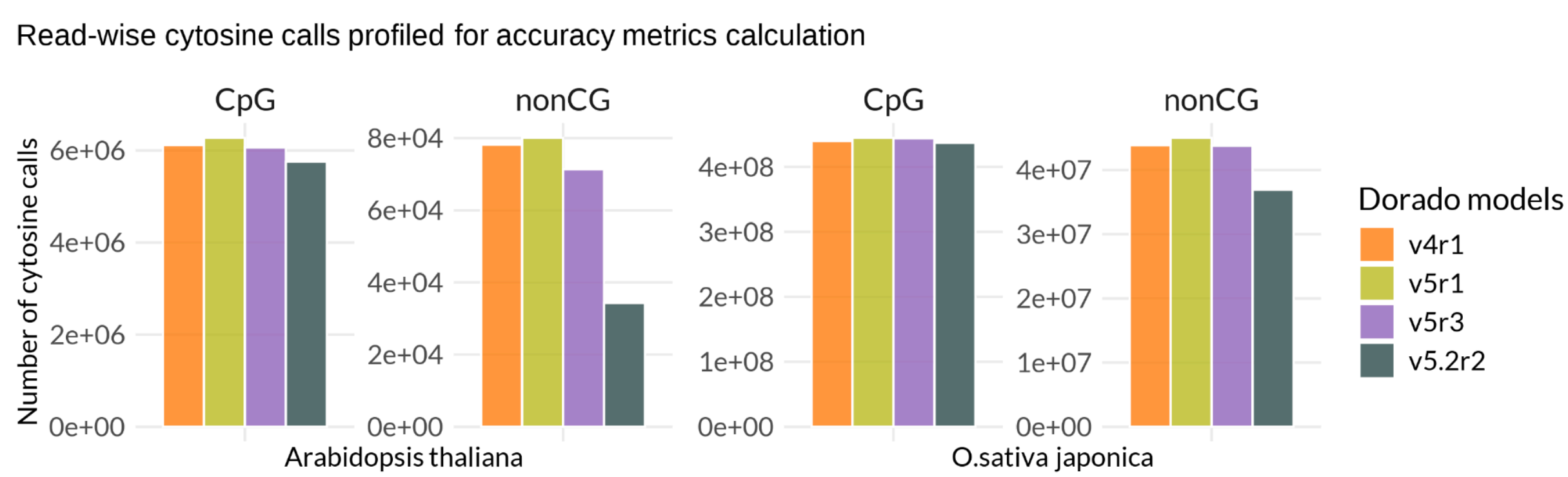

Sincerely,

Divya Tej Sowpati

Response to Reviewer Comments

Reviewer #1 (Remarks to the Author)

0 & 10D) Read the workflow output this round. Final files in output/meta/{tool}/ are methylBEDs with metadata columns — useful, but not a benchmark. The scoring lives in performance_metrics_full_data.sh, which states: "This is a scaffold script... not intended to be used as is. The variables need to be adapted." Below that are awk one-liners with hardcoded column indices, motif by motif. Figures come from 12 bespoke R scripts.

So there's no reusable scoring layer. To benchmark Dorado v6 next quarter, someone has to rewrite that scaffold. That's why the contribution is limited — not because the data isn't valuable, but because the benchmark part doesn't exist as a tool.

What I strongly suggest the authors should do - have the output from the nextflow not just be the methylBED but also F1/precision/recall/RMSE per tool/motif/context. Your awk is 90% of it. Refactor it into something reusable and this becomes a much stronger community resource.

Response: We thank the reviewer for their in-depth analysis and comment. We completely agree that code reusability is essential to serve the community, and explain below how our work achieves that.

There are three distinct parts to address in this comment.

Regarding hardcoded metrics and a reusable scoring layer
The specific script the reviewer examined was for the explicit purpose of allowing users to exactly reproduce the results presented in our manuscript. For that specific goal of strict reproducibility, it functions as intended. However, we agree with the reviewer's broader point that a generalized, reusable scoring layer is important. To this end, we provide a standalone R script (available in the GitHub repository at benchmark_nextflow/bin/**methylation_metrics.R**). This script accepts a methylBed file, ground truth file, and a motif as command-line arguments, and automatically calculates the performance metrics the reviewer highlighted (F1, precision, recall etc.). Furthermore, because our scripts generate and process the standardized methylBed output format from modkit, they are robust and **agnostic to future dorado updates** (e.g., v6 and beyond).

Regarding including metrics calculation in the same nextflow pipeline:
Our Nextflow pipeline intentionally terminates at methylBed generation and annotation, decoupling the accuracy metrics and other downstream analysis into separate scripts. This design choice optimizes computational efficiency and resource management. The methylBed generation is highly compute-intensive and requires GPU acceleration, typically run in an HPC environment or on specialized cloud instances. In contrast, downstream metric calculations and visualization are lightweight, CPU-based tasks that often require interactive tuning.

Even on cloud platforms like AWS, it is far more cost-effective to run the Nextflow pipeline on a transient, high-performance GPU instance, and subsequently download the methylBed files to run the downstream analysis locally or on cheaper, low-vCPU instances. Therefore, we maintain that **keeping these steps separate provides the most practical, convenient, and cost-effective** framework for users.

Regarding individual R scripts for figures
The reviewer correctly notes the presence of several individual R scripts. These are intentionally provided as standalone scripts to allow users to exactly reproduce the specific

figures presented in the manuscript. We find that maintaining modular, distinct scripts improves code readability and makes it easier for users to adapt individual visualization steps for their own datasets, rather than navigating a single monolithic codebase.

3) Agree to disagree. The authors are clear on what they are doing.

Response: We thank the reviewer for their understanding and for their thorough and thoughtful evaluation of our manuscript.

4a) I acquiesce on this point, I still feel like given the topic this could//should have been expanded about the window of signal the basecaller is using.

Response: We agree with the reviewer that this is an important aspect of the topic. We would like to direct the reviewer's attention to **lines 375–379** of the reviewed manuscript, where we have already included a discussion regarding the signal window utilized by Dorado to provide this relevant context for the readers.

9b) Still not addressed. State the threshold and coverage you used to call locations "fully methylated" in HG002, mouse brain, and mouse ESC. Composite datasets don't answer it.

Response: The details of threshold and coverage (100% methylated, >20x depth) used to call fully methylated locations is provided in Methods, section "Calculating F1 scores", **line 1053** onwards. The number of such locations for all datasets, including the three datasets that the reviewer mentioned, tool and context wise, is provided in **Table S7** (now called **Supplementary Data 7**). This has also been clarified and summarized in Results section, **lines 231-236**.

10B) Ok

Response: We thank the reviewer for their confirmation.

10C) The FP/FN bar chart is good. Please add this to the supplement.

Response: Thank you. This figure has been added to the supplementary information (Supplementary Figure S11b) and cited appropriately (**lines 374-375**).

20B) The Qscore plot answers the question - make sure this is clear and please add this to the supplement.

Response: Thank you. This figure has been added to the supplementary information (Supplementary Figure S26) and cited appropriately (**lines 673-678**).

Sincerely,

Divya Tej Sowpati
